# Sequential Phage Delivery Can Outperform Cocktails by Delaying Cross-Resistance Evolution

**DOI:** 10.3390/v18040404

**Published:** 2026-03-25

**Authors:** Elizabeth C. Stuart, Justin R. Meyer

**Affiliations:** Department of Ecology, Behavior and Evolution, University of California San Diego, La Jolla, CA 92093, USA; estuart@ucsd.edu

**Keywords:** bacteriophage therapy, sequential delivery, phage cocktails, resistance evolution, cross-resistance, *Escherichia coli*, experimental evolution

## Abstract

Antimicrobial resistance has renewed interest in bacteriophage therapy, yet bacterial evolution frequently undermines treatment efficacy. Combination phage therapy is commonly implemented as simultaneous phage cocktails, but whether this is optimal remains in question. Here, we experimentally compared simultaneous versus sequential administration of two phages, an evolved λ called ‘λtrn’ and T2, on *Escherichia coli* K-12 under controlled laboratory conditions. Across replicated experiments, treatment outcome depended strongly on delivery strategy, dosing order, and timing. Contrary to expectations, sequential delivery consistently achieved greater and more sustained bacterial suppression than simultaneous cocktails, although only when T2 initiated the sequence. Phenotypic assays revealed that treatment differences were driven by the accessibility and timing of cross-resistance evolution. λ-first treatments rapidly selected for cross-resistant bacteria prior to exposure to the second phage, rendering subsequent treatment ineffective. In contrast, T2-first sequential treatments delayed or limited cross-resistance and frequently produced single-phage resistance or collateral sensitivity. Cocktail treatments showed intermediate dynamics, with cross-resistance evolving more slowly but consistently. Whole genome sequencing identified distinct genetic routes to cross-resistance, including regulatory mutations in *envZ* affecting expression of the phage receptor OmpF, as well as envelope-modifying, mucoidy-associated mutations. Engineering *envZ* mutations into unevolved backgrounds confirmed the mutation’s sufficiency to confer low-cost cross-resistance. Together, these results demonstrated that phage therapy efficacy depended not only on phage composition but on how selection pressures were ordered in time, highlighting evolutionary steering as a powerful principle for multi-phage therapy design.

## 1. Introduction

Antimicrobial resistance (AMR) is a global health crisis. Recent estimates attribute 1.27 million deaths directly to bacterial AMR in 2019, with 4.95 million deaths associated with AMR overall [1]. Forecasts suggest AMR-associated mortality could rise substantially by 2050, reaching 8.22 million deaths, including 1.91 million directly attributable deaths [2]. This burden has renewed interest in bacteriophage (phage) therapy as an alternative or complement to antibiotics. However, as with antibiotics, therapeutic efficacy can be undermined by rapid bacterial evolution: resistance to therapeutic phages has been reported in approximately three-quarters of clinical studies in which resistance emergence was monitored [3].

A central strategy for limiting resistance evolution in phage therapy is combination treatment, most commonly implemented as simultaneous multi-phage regimens or “cocktails.” Because systematic comparisons of phage delivery strategies—such as cocktails versus sequential administration—remain limited, much of our conceptual framework comes from the extensive literature on antibiotic combination therapy, where the evolutionary consequences of combination strategies have been studied. In antibiotics, combination regimens deploy two or more drugs with distinct modes of action to broaden activity and, in principle, reduce the probability that resistance evolves via a single mutation [4,5,6]. However, antibiotic studies have shown that combinations can have opposing evolutionary outcomes: some suppress resistance evolution [6,7], whereas others accelerate it [7,8] or select for broadly resistant genotypes via generalized resistance mechanisms [8,9].

A key determinant of these outcomes is whether resistance mechanisms generate cross-resistance or collateral sensitivity. Cross-resistance can arise through regulatory or physiological changes (e.g., increased efflux or envelope remodeling) that simultaneously reduce susceptibility to multiple agents [10,11], thereby undermining the efficacy of combination treatments. In contrast, collateral sensitivity—where adaptation to one agent increases susceptibility to another—can make combination or ordered (sequential) strategies unusually potent. Large-scale mapping of resistance interactions across antibiotics has revealed structured networks of cross-resistance and collateral sensitivity in *E. coli* [12], and additional experiments demonstrate that exploiting collateral sensitivity through sequential exposure can strongly constrain evolutionary paths to multidrug resistance [13]. Together, this body of work provides a framework for anticipating when multi-phage cocktails may inadvertently select for broadly resistant bacterial genotypes, and when sequential phage delivery may instead bias evolutionary trajectories in ways that prolong suppression and limit resistance evolution [12,13,14].

Combination therapy is standard practice in clinical phage applications, most often implemented as simultaneous phage “cocktails.” In practice, cocktails are frequently motivated by pragmatic coverage—maximizing the chance that at least one phage is active against a patient isolate—while also being assumed to impede resistance by requiring bacteria to evade multiple phages. However, simultaneous exposure can also select for generalized resistance mechanisms (e.g., broadly protective envelope changes or other host defense pathways) that confer cross-resistance across taxonomically diverse phages, potentially limiting the intended benefit [15]. In a recent analysis of 100 consecutive personalized phage therapy cases in Belgium, cocktails were used more often than monophage therapy (61 vs. 39 cases), yet clinical improvement rates were similar between approaches (78% for cocktails vs. 82% for single-phage therapy) [16]. While resistance evolution was not tracked in that study, controlled comparisons in other systems have shown that cocktails can delay resistance and improve suppression relative to monophage treatment in some contexts [17,18]. These mixed outcomes highlight the need to identify when multi-phage strategies constrain resistance and when they inadvertently enrich broadly resistant bacterial genotypes.

Beyond simultaneous cocktails, phages can also be deployed sequentially. Sequential delivery changes selection pressures over time and can outperform cocktails even without collateral sensitivity. If cross-resistance mutations are rare relative to single-phage resistance mutations, the first phage may drive the bacterial population toward resistance that is largely specific to that phage; introduction of a second phage can then strongly reduce the population before a second, independent resistance mutation arises, extending the overall period of suppression. In the most favorable case, sequential delivery can additionally exploit collateral sensitivity, where resistance to the first agent increases vulnerability to the second—an effect shown to constrain evolutionary paths to multidrug resistance in antibiotic treatments [19]. Thus, the relative merits of cocktails versus sequential delivery depend critically on (i) the relative mutational accessibility of cross-resistance compared to mono-resistance, (ii) the degree of positive or negative resistance correlations, and (iii) the demographic consequences of resistance mutations.

To date, direct comparisons of cocktail versus sequential phage delivery remain limited. Two studies in *Pseudomonas aeruginosa* evaluated these approaches and underscored the importance of receptor usage, order, and timing. Hall et al. compared multiple delivery schemes (single phages, paired and multi-phage cocktails, and sequential regimens) and found that sequential delivery could match cocktail efficacy in some cases, with no consistent advantage for either strategy in delaying resistance [20]. Wright et al. further showed that resistance outcomes depended on the timing and order of exposure: when phages shared a receptor, resistance to the first often conferred cross-resistance to the second, eroding sequential benefits, whereas sequential exposure to phages using different receptors could yield distinct resistance costs and strengths, and sometimes asymmetric cross-resistance across exposure order [21]. Collectively, these studies suggest that optimal delivery strategy cannot be predicted without information on the genetic basis of resistance and the degree to which resistance to one phage alters susceptibility to the other.

Although these questions are ultimately clinical, mechanistic progress requires controlled experimental systems in which resistance evolution can be tracked and linked to genetic causes. Here, we use laboratory evolution to test how multi-phage administration strategy—simultaneous cocktail versus sequential exposure—shapes bacterial suppression and the evolution of resistance. We focus on *Escherichia coli*, a highly relevant species in the AMR crisis, including WHO-designated priority resistant phenotypes such as third-generation cephalosporin-resistant and carbapenem-resistant *E. coli* [22]. While our experiments use the non-pathogenic laboratory strain *E. coli* K-12, it provides unmatched laboratory resources for dissecting evolutionary dynamics and resistance mechanisms with precision [23].

A central feature of our design is that the focal phages are multi-receptor generalists (T2: OmpF and FadL, and λ*trn*: OmpF and LamB), which can impose a higher barrier to resistance evolution than single-receptor phages. Resistance to phages often arises through mutations altering surface structures and outer-membrane receptors; when a phage can use multiple receptors, complete resistance may require multiple mutations and can carry substantial fitness costs [24]. This property has two opposing implications for multi-phage therapy. On the one hand, multi-receptor phages can be intrinsically suppressive because resistance is rarer and costlier, potentially reducing the incremental benefit of adding additional phages in a cocktail, or they may even promote cross-resistance if resistance to one multi-receptor phage is mediated by broad envelope remodeling or other generalized mechanisms, thereby diminishing efficacy. On the other hand, resistance to a multi-receptor phage may proceed via *partial* receptor loss or cause physiological trade-offs, resulting in bacterial genotypes that remain susceptible, or even become hypersensitive, to additional phages in a cocktail. These considerations make multi-receptor phages a stringent and clinically relevant test case for evaluating whether sequential delivery can outperform cocktails, and for identifying the evolutionary and mechanistic conditions under which cocktails may underperform despite their widespread use.

Using this framework, we quantify how delivery strategy affects bacterial suppression, test the influence of the starting phage and dosing interval in sequential regimens, and measure the extent to which resistance and cross-resistance explain treatment outcomes. Contrary to the common expectation that cocktails provide the strongest protection against resistance, we find that cocktail treatment can underperform a sequential regimen depending on the order of exposure. Patterns of suppression implicate cross-resistance as a primary driver of these differences. We support this inference by directly assaying resistance phenotypes of evolved isolates and by mapping the genetic basis of resistance using whole genome sequencing and targeted genetic tests, which together reveal the molecular mechanisms underlying cross-resistance and other resistance routes. These results provide an experimentally grounded framework for predicting when sequential phage delivery should outperform cocktails and highlight cross-resistance as a key limiting factor in multi-phage therapy design.

## 2. Materials and Methods

### 2.1. Bacterial Strains and Phages

To study how phage delivery strategy influences the efficacy of multi-phage treatments, we used *Escherichia coli* K-12 strain BW25113 (WT) [25] and two lytic bacteriophages: λ*trn* and enterobacteria phage T2 (provided by Jyot Antani, Yale University New Haven, Connecticut). Phage T2 was a dual receptor using phage, its receptors were *fadL* [26] and *OmpF* [27]. Phage λ*trn* was a descendant of λ cI26, an obligatory lytic mutant that was previously coevolved with *E. coli* REL606 for 28 days and has been shown to be much more suppressive than typical lab strains [24,28,29]. λ*trn* was also a dual receptor phage, it shared one receptor with T2 (OmpF) and had one unique receptor (LamB) [24].

We confirmed their receptor use by spot assays on knockout hosts. We spotted 2 μL of phage lysate onto lawns of ~10^8^ *E. coli* cells. Phage were spotted onto wild-type (WT)*,* LamB^–^ OmpF^–^ *E. coli,* and FadL^–^ OmpF^–^ *E. coli.* T2 was unable to form plaques on FadL^–^ OmpF^–^ *E. coli,* and λ*trn* was unable to form plaques on LamB^–^ OmpF^–^ *E. coli.* We also plated on all three single receptor gene knockouts to double check that a single receptor deletion was not sufficient to block either phage.

### 2.2. Initial Cocktail Versus Sequential Experiment

To determine how phage administration strategy (cocktail versus sequential delivery) affects bacterial suppression, we compared monophage treatments, simultaneous phage cocktails, and sequential phage delivery using λ*trn* and T2. Experiments were conducted in liquid culture over 14 days.

Replicate populations were propagated in 36 mL culture tubes containing 4 mL of Tris-LB medium (0.28 g K_2_HPO_4_, 0.08 g KH_2_PO_4_, 1 g (NH_4_)_2_SO_4_, 10 g tryptone, and 5 g yeast extract per liter of water supplemented to a final concentration of 50 mM Tris (pH 7.4), 0.2 mM CaCl_2_, and 10 mM MgSO_4_) [28]. Cultures were inoculated with approximately 9.0 × 10^4^ *E. coli* colony-forming units (CFUs) and ~10^6^ plaque-forming units (PFUs) of phage, creating a multiplicity of infection (MOI) of ~10. Cocktail treatments were initiated with a combined total of ~10^6^ PFUs, with T2 making up half of the total number phage and λ*trn* making up the remainder (~5.0 × 10^5^ PFU each). Tubes were used rather than more convenient plates to propagate clinically relevant population sizes of bacteria.

For sequential treatments, populations received an initial dose of either T2 or λ*trn*, followed by a second dose consisting of the alternate phage. For cocktail treatments, phages were administered simultaneously from a mixed stock containing equal numbers of λ*trn* and T2 particles. Monophage treatments received either one or two doses of a single-phage species, depending on treatment group (Table S1).

Replications differed by the treatment type. Sequential treatments were initiated with six replicate populations, cocktail treatments with three replicates, and monophage treatments with three replicates per condition. Three replicate populations without phage exposure served as no-phage controls. Altogether this represented an extensive survey with 72 independent trials (Table S1).

Cultures were incubated at 37 °C with shaking at 220 rpm. Every 24 h, 40 µL (1%) of each culture was transferred into 4 mL of fresh Tris-LB medium. Transfers were performed daily for the duration of the experiment. Prior to each transfer, samples were collected to estimate bacterial density and to assay phage presence. Samples were archived in 15% (*v*/*v*) glycerol and stored at −80 °C.

### 2.3. Administration of Second Phage Doses

Second phage doses were administered on one of three schedules: Early (24 h), Mid (48 h), or Late (72 h) following the initial phage exposure. Second doses were added immediately after daily transfers and sampling, and prior to incubation.

Sequential and monophage treatments received a second dose of a single-phage (~10^6^ PFU of either T2 or λ*trn*, depending on treatment). Cocktail treatments received ~10^6^ PFU total from a mixture of the two phage stocks (Table S1).

### 2.4. Estimation of Bacterial Density

Bacterial density was estimated daily by measuring optical density at 600 nm (OD_600_) using a Tecan Sunrise microplate reader. For each population, samples were loaded into two independent 96-well plates with 200 µL per well, and OD measurements were averaged across plates. No-phage controls were measured in parallel. Optical density (OD) and colony-forming unit (CFU) counts were extensively compared in a representative experiment and showed a strong relationship (Table S17). As a result, OD was used for most bacterial density measurements to reduce time and resource demands.

### 2.5. Phage Detection by Spot Assay

Daily spot assays were used to detect the presence of phage. One milliliter of each culture was centrifuged at maximum speed for 1 min, and 2 µL of the clarified supernatant was spotted onto lawns of ~10^8^ WT *E. coli* prepared in molten soft agar (10 g tryptone, 1 g yeast extract, 8 g sodium chloride, 7 g agar, 1 g glucose, 1.23 g magnesium sulphate per L water) over LB agar plates. Plates were incubated at 37 °C for 24 h. Clearing or plaque formation was scored as phage presence.

Additionally, cryopreserved samples were periodically revived and assayed for the presence of T2 or *λtrn* by plating on double-knockout bacterial strains. T2 infected strains lacking OmpF and LamB and not those lacking OmpF and FadL, whereas *λtrn* exhibited the opposite infection pattern. Knockout strains were generated and previously described in Borin et al. 2023 [30]. See Supporting Information Figures S1–S6 for additional experimental details and verification of strain fidelity across treatments.

### 2.6. Follow-Up Sequential Versus Cocktail Experiments

Based on the results from the initial experiment, follow-up studies focused on treatments showing the largest differences in suppression and, of particular interest, T2-initiated sequential treatments and cocktail treatments under mid and late dosing schedules (Table S1). Two follow-up experiments were conducted, lasting 5 days and 14 days. Replications were increased to 12 replicate populations per treatment to improve statistical power. Culturing, transfer, dosing, sampling, and storage procedures were identical to those used in the initial experiment. No-phage controls were included with three replicates.

### 2.7. Bacterial Isolation from Frozen Communities

To isolate bacteria free of phages, frozen samples were streaked onto LB agar plates supplemented with 5 mM of sodium citrate to inhibit phage infection. Sodium citrate can act as a chelating agent, binding the calcium needed for phage infection [31]. Plates were incubated at 37 °C for 24 h. Three colonies per replicate population were randomly selected and restreaked twice on fresh LB citrate plates. A representative colony was then used to inoculate 4 mL of 10 mM sodium citrate LB Lennox medium for downstream assays.

### 2.8. Bacterial Survival (Resistance) Assays

Bacterial survival following phage exposure was assayed to quantify resistance evolution. WT *E. coli* served as a positive control. Cultures were grown overnight in 10 mM sodium citrate LB Lennox at 37 °C. Forty microliters of overnight culture were inoculated into 4 mL Tris-LB and grown for 2 h. For each isolate that was tested, there were three treatment groups: no-phage, T2-phage, and λ-phage.

Phage treatments received 100 µL of ~10^9^ PFU phage lysate and were incubated for 30 min at 37 °C. Phage infections were halted by placing cultures on ice and adding 40 µL of 1 M sodium citrate. Sodium citrate helped to prevent further phage infection by acting as a chelating agent binding to the calcium needed for phage infection [31]. Serial dilutions were plated to estimate surviving bacterial density.

### 2.9. MAGE of envZ

To test whether the *envZ* mutation identified in a λ-first isolate (λ-first day 1 L3.2) is sufficient to confer cross-resistance, we engineered this single mutation (3,529,094, T→G, Q45P (CAG→CCG)) into a *mutS*^−^ *E. coli* K-12 [25] background using multiplex automated genome engineering (MAGE) [32]. We extracted the λ-Red by recombineering plasmid pKD46 from (HWEC106 provided by Harris Wang, Columbia University, New York, NY, USA) and electroporating (~1.8 kV, 25 µF capacitance, and 200 Ohms resistance in 0.1 cm cuvettes) it into the *mutS*^−^ *E. coli.* Recombineering was induced with 20 µL of 1 M arabinose (1 h incubation). A 90 nt single-stranded oligonucleotide (TCGGTCATCAACATACGCACTTCGTACGCGAGGACTTTATTAAACgGCTGGAGGCTCGGCAAAATCGCGAAGTTCAGCACCACCAGATAA) targeting *envZ* (designed using MODEST [33]) was electroporated into three independent replicate cultures (plus a no-oligo negative control), and cells were cycled through five rounds of MAGE. Three colonies from each of the three replicates were screened for the target mutation. Because initial screening yielded no edited colonies, we added an enrichment step by challenging post-MAGE cultures with λ*trn* prior to plating. We cultured ~10^7^ cells in 3 mL of Tris-LB with 4 µL of 50 ng/mL carbenicillin at 30 °C for 2 h. After 2 h we added 1 mL of ~10^10^ λ*trn* lysate (MO1 ~100) and incubated for an hour at 30 °C. This enrichment step increased recovery of the edited clones from 0/9 unscreened colonies to 9/9 screened colonies with the target mutation. Candidate colonies were screened by PCR and Sanger sequencing of *envZ*, using *envZ* forward primer (5-GGC TGG TCC GAA ACT GTA AT-3′) and reverse primer (5′-CTG CAT CGA CGT GCA GAT TT-3′). One confirmed single-mutation clone from each replicate was retained for downstream phenotyping.

### 2.10. Resistance Coefficient Calculation

Resistance coefficients (RC) were calculated to quantify the relative susceptibility of bacterial isolates to phage infection compared to WT *E. coli*. Bacterial densities were measured after 30 min incubation in the presence (P) or absence (NP) of phage, and RC was defined as:

$$RC=\frac{ln(\frac{WT_{P}}{WT_{NP}})-ln(\frac{Iso_{P}}{Iso_{NP}})}{ln(\frac{WT_{P}}{WT_{NP}})}$$

where WT_p_ is the colony-forming bacterial density of WT *E. coli* following phage exposure; WT_NP_ is the bacterial density of WT *E. coli* in the absence of phage; Iso_NP_ is the bacterial density of the tested bacterial isolate without phage exposure; and Iso_p_ is the bacterial density of the same isolate following phage exposure.

Log transformation was used because bacterial growth and phage infection were exponential processes that can be approximated over short intervals by *dN/dt = rN − ϕPN*, yielding *N(t) = N(0)e (r − ϕP)t*. The log ratio *ln(N_P_/N_NP_)* therefore isolated the effective per capita killing term and removed variation attributable to intrinsic growth rate. Using log-transformed ratios linearized multiplicative dynamics and reduced heteroscedasticity inherent to CFU measurements. The RC metric comprised three conceptual components: First, *ln*(*Iso_P_/Iso_NP_*) quantified the net effect of phage exposure on the isolate while correcting for isolate-specific growth during the assay. Second, *ln*(*WT_P_ /WT_NP_*) provided a day-specific benchmark of phage killing efficiency against WT bacteria, controlling for variation in assay performance across the experimental days. Third, normalizing the difference between the isolate and WT killing by the WT benchmark yielded a dimensionless measure of relative resistance that was robust to the day effects and variation in phage titer. Under this formulation, RC ≈ 0 indicated WT-like susceptibility, RC > 0 indicated resistance, and RC < 0 indicated hypersusceptibility.

The assay duration was limited to 30 min to capture early infection dynamics prior to substantial phage amplification or resistant mutant expansion, thereby approximating the immediate susceptibility phenotype rather than longer-term coevolutionary outcomes.

### 2.11. Growth Rate Analysis

Isolated bacteria were acclimated to Tris-LB conditions over two successive overnight passages before growth rate measurements. Overnight cultures were diluted by transferring 40 µL into 4 mL of Tris-LB (~10^7^ cells), 200 µL samples were then transferred to 96-well plates and monitored spectrophotometrically. The optical density was measured every 5 min for 24 h. Maximum growth rates were estimated using the easyliner function of the growthrates r package [34] which fitted segments of linear models to the log-transformed data and identified the maximum growth rate.

### 2.12. Statistical Analysis and Plotting

All statistical analyses and data visualization were performed in RStudio [35]. All figures were generated using the ggplot2 package [36]. Statistical modeling and analyses were conducted using the base stats package [37], and model evaluation and selection were performed with the performance package [38].

In the initial experiment, the bacterial densities among treatments were compared using Mann–Whitney U tests. These comparisons were performed on the bacterial densities measured 24 h after the second phage dose. For regression analyses, the treatment effects were modeled using the mean bacterial density for each replicate population averaged across the full 14-day experiment. Multilinear regression models were run using Rstudio version 4.5.2 with the base statistics package, and model selection was done using the performance package, which calculated AIC, R^2^, and R^2^_adj._

Data deviated from normality and attempts to normalize residuals using Box–Cox transformations were unsuccessful. Therefore, rank-transformed data were used for all regression models. Statistical significance was assessed using a threshold of *p* < 0.05. Resistance coefficient data were reported as mean ± 95% confidence intervals (CI).

### 2.13. Bacterial Whole-Genome Sequencing

We sequenced bacterial isolates from the initial suppression experiment. Genomic DNA was extracted using Invitrogen™ PureLink™ Genomic DNA Mini Kit. All isolates, except T5.3, were sequenced by SeqCenter (Pittsburgh, PA, USA), while T5.3 was sequenced by Plasmidsaurus (San Diego, CA, USA). Mutations were called using Breseq version 0.39.0.

## 3. Results

### 3.1. Initial Suppression Experiment

To evaluate how the phage delivery strategy influenced bacterial suppression and resistance evolution, we simulated a bacterial infection under controlled laboratory conditions by propagating *Escherichia coli* K-12 populations in liquid cultures for 14 days with daily passages to maintain growth conditions. During this period, replicate bacterial populations were subjected to different phage-treatment regimens, allowing us to compare bacterial population dynamics and treatment efficacy across strategies.

We first compared single-phage (monophage) treatments to multi-phage treatments. Multi-phage treatments were implemented either as simultaneous cocktails, in which both phages were either administered together or as sequential regimens in which phages were administered one after the other. In sequential treatments, populations received an initial dose of either *λtrn* or T2, followed by a second dose consisting of the alternate phage. In contrast, cocktail treatments received both phages at each dosing event. For both cocktail and sequential regimens, the timing of the second dose was varied systematically, with doses administered early (24 h), mid (48 h), or late (72 h) following the initial exposure.

We quantified treatment efficacy by measuring bacterial density daily via optical density at 600 nm (OD_600_), where lower OD values indicated stronger bacterial suppression and improved treatment efficacy. These daily measurements were used to construct a multilinear regression model assessing the effects of treatment strategy (monophage, cocktail, or sequential), the starting phage identity, and the timing of the second dose on the bacterial density. For each replicate population, we used the mean OD_600_ across the full 14-day experiment as the response variable.

The model selection was based on AIC, and adjusted $R^{2}$ identified a best-fit model (AIC = 482; $R_{adj}^{2}=0.77$) in which the treatment strategy (*p* = 0.005), the starting phage identity (*p* = 3.29 × 10^−15^), the interaction between the treatment strategy and starting phage (*p* = 0.014), and the three-way interaction between the treatment strategy, timing, and starting phage (*p* = 0.017) all had significant effects on the bacterial density (Table S2).

These patterns persisted when the analysis was restricted to multi-phage treatments only, indicting that the treatment strategy effect was not driven entirely by the comparison between single- and multi-phage treatment but also between different multi-phage administrations (Table S3). In this subset, both the delivery strategy (cocktail versus sequential; *p* = 0.007) and starting phage identity (T2, *λtrn*, or both; *p* = 1.09 × 10^−12^) significantly influenced the bacterial density. Interactions between the timing and treatment strategy (*p* = 0.06) and between the timing and starting phage identity (*p* = 0.068) did not meet our threshold for statistical significance, but they suggested weak trends consistent with timing-dependent effects.

To identify which specific treatment regimens most effectively suppressed the bacterial populations, we conducted pairwise comparisons among treatments informed by the global regression analysis. Because pairwise tests required correction for multiple comparisons and therefore had reduced statistical power, we focused on a biologically and statistically informative time point: the bacterial density measured 24 h after the second phage dose. This time point captured treatment efficacy after both phages had been applied while phage-mediated suppression remained strong, maximizing the detectable differences among treatment strategies.

**Figure 1:**
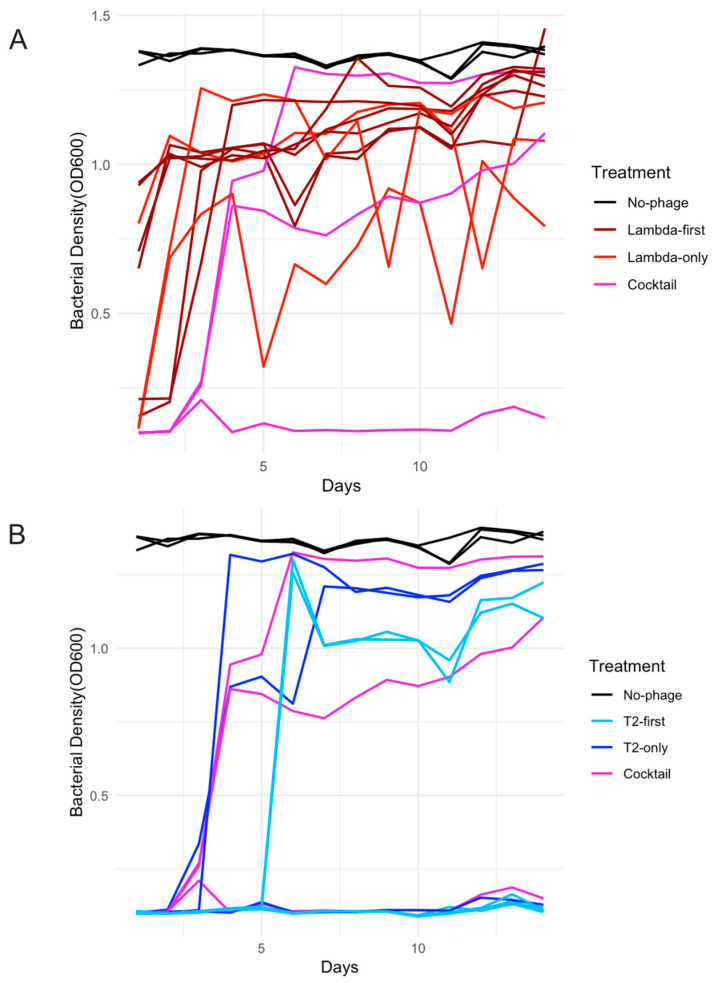
Subset of bacterial population dynamics from initial suppression experiments illustrating variation across phage treatments. Bacterial density (OD_600_) was measured once daily for two weeks across multiple phage suppression assays and a no-phage control. (**A**) λ-first sequential treatment, λ-only monophage treatment, and cocktail treatment. (**B**) T2-first sequential treatment, T2-only monophage treatment, and cocktail treatment. All data shown correspond to late dosing regimens, in which phages were added at the start of experiments and again on day 3.

The first and most immediate observation is that λtrn—even though it was an evolved dual-receptor phage—did not maintian suppression under these experimental conditions. In several replicates, λtrn alone lost suppressive capacity within one day, and in all replicates by day three (Figure 1A). This contrasted with a previous study conducted in a minimal (rather than rich) medium and using a different laboratory strain*, E. coli B* REL606, where bacterial suppression was maintained for up to three weeks [24]. This discrepancy highlighted the contingency of phage therapeutic outcomes and underscored the importance of identifying the ecological and evolutionary drivers of successful suppression. T2, on the other hand, was signficanty more suppressive than λtrn (*p* = 0.022, Table S4), as the bacterial densities remained below the limit of optical detection for at least three days in all T2-only replicates, and even for the entire duration of the experiment in some replicates (Figure 1B).

Next, we explored whether the combination treatments were more effective. The sequential treatment with λ administered first, ‘λ-first treatment’, showed indistinguishably poor supression from λ-alone (0.519; Table S6, Figure 1A). This was surprising, because when T2 was administered without the cells first being exposed to λtrn, T2 supressed the bacteria, but now it appeared to have no effect. We hypothesized that λtrn selected for resistant mutations that confered cross-resistance to T2, interfering with T2’s ability to supress the bacteria. We directly tested this hypothesis in Section 3.3.

Analyses of treatments in which T2 was administered alone or in combination revealed a marked contrast (Figure 1B). Unlike the λ-only or λ-first treatments, many replicates in the T2 treatments declined below detection limits and remained there, consistent with bacterial extinction. Direct plating confirmed that bacteria were eliminated in several replicates. Moreover, when T2 was administered initially—either alone or as part of the cocktail—bacterial populations were suppressed for at least three days. The T2-only and cocktail treatments displayed nearly identical dynamics, indicating that T2 dominated the interaction when both phages were introduced simultaneously (*p* = 0.789; Table S6, Figure 1B). However, in the T2-first treatment, where λtrn was introduced two to three days after T2, suppression was further prolonged, indicating that λtrn can enhance suppression when applied sequentially but not when administered concurrently (*p* = 0.004, *p* = 0.024; Table S5, Figure 1B). This pattern suggested that bacteria may evolve resistance to T2 during the initial three days but do not acquire cross-resistance to λtrn, revealing a possible asymmetry in cross-resistance evolution between the two phages. This hypothesis was directly tested in Section 3.3.

Contrary to the expectation that simultaneous phage cocktails provided the strongest suppression, the sequential treatments initiated with T2 outperformed the cocktail treatments. In both dosing regimes where significant differences were detected, the T2-started sequential treatments achieved lower bacterial densities than the cocktails (Mid: *p* = 0.004; Late: *p* = 0.024; Table S5). These results demonstrated that sequential delivery can exceed the efficacy of cocktails, but that this advantage depended critically on the identity of the starting phage.

**Figure 2:**
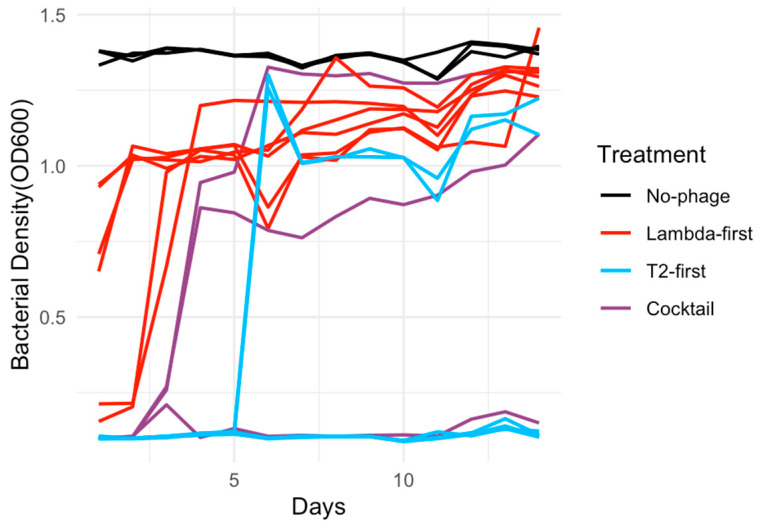
Subset of bacterial population dynamics from initial suppression experiments illustrating variation across phage treatments. Bacterial density (OD_600_) was measured once daily for two weeks across multiple phage suppression assays and a no-phage control. λ-first and T2-first represent opposite sequential treatment orders, while cocktail treatment included both phages at each dosing event. All data shown correspond to late dosing regimens, in which phages were added at the start of experiments and again on day 3.

This pattern contrasted sharply with sequential treatments initiated with *λtrn*. Across all timing regimes, *λ*-first sequential treatments performed poorly and were consistently outperformed by both the cocktail treatments and T2-first sequential regimens. Direct comparisons confirmed that the sequential treatments initiated with T2 significantly outperformed those initiated with *λtrn*, regardless of the timing of the second dose (early: *p* = 5.0 × 10^−4^; mid: *p* = 0.005; late: *p* = 0.005; Table S6). Differences among treatments were most pronounced under the late dosing regimen (Figure 2), where the starting phage identity exerted the strongest influence on bacterial suppression.

### 3.2. Follow-Up Experiments Confirm Superior Performance of T2-Initiated Sequential Treatments

**Figure 3:**
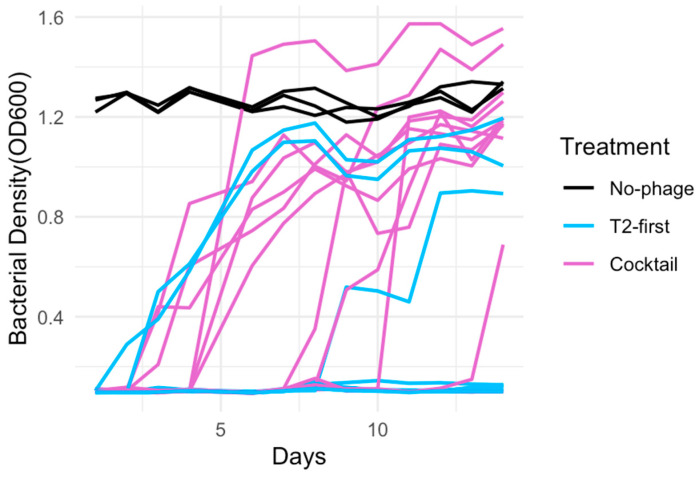
Sequential treatments outperformed cocktail treatments. Bacterial density was measured with a spectrophotometer at wavelength 600 nm once a day for two weeks. All data were for the late dosing regimens, with phages added initially and on day 3.

To further evaluate these counterintuitive results—specifically, that sequential delivery can outperform simultaneous cocktails—we conducted follow-up experiments focusing on the treatment comparisons that showed the largest differences in the initial study and yielded the most surprising results. We restricted these experiments to the T2-initiated sequential treatments and cocktail treatments, under the mid and late dosing schedules. Two follow-up experiments were performed: one lasting 5 days and a second lasting 14 days. In both follow-up studies, we increased replication to improve statistical power. The number of replicate populations were increased from 6 to 12 for the sequential treatments and from 3 to 12 for the cocktail treatments (Table S7). Across both experiments, the pattern observed in the initial study was reproduced: the T2-initiated sequential treatments consistently outperformed the cocktail treatments. We found no significant differences among the three experimental runs (Table S8) (*p* = 0.465). We assessed the performance of the treatments based on the average bacterial density (OD600) per replicate across the 14-day experiment. In the 5-day experiment, the T2 sequential treatments achieved significantly lower bacterial densities than the cocktails under both mid and late schedules (mid: *p* = 0.019; late: *p* = 0.006). In the 14-day experiment, this difference remained significant under the late dosing regimen (*p* = 0.035; Table S9; Figure 3).

We further analyzed the follow-up data using regression models analogous to those applied in the initial experiment. In both the 14-day (AIC = 479; $R^{2}=0.53$; $R_{adj}^{2}=0.5$) and 5-day (AIC = 384.1; $R^{2}=0.26$; $R_{adj}^{2}=0.21$) datasets, the treatment strategy (sequential versus cocktail) had a significant effect on the bacterial density (14-day: *p* = 3.04 × 10^−9^; 5-day: *p* = 3. × 10^−4^). In the 14-day follow-up experiment, we also detected a significant interaction between the treatment strategy and dosing timing (*p* = 0.03; Table S10), indicating that the relative advantage of sequential delivery depended on when the second phage was administered.

### 3.3. Patterns of Resistance and Cross-Resistance Evolution

Next, we investigated why treatment efficacy differed among the sequential and cocktail strategies. Our leading hypothesis was that these differences arose from variations in the propensity of each treatment to select for cross-resistant bacterial genotypes. Specifically, if mutations conferring resistance to both phages were more readily accessible than λ*trn*-specific resistance, then λ-first sequential treatments would have rapidly selected for cross-resistant bacteria, leading to an early treatment failure. In contrast, if T2-specific resistance was more common than cross-resistance, the T2-first sequential treatments would have initially selected for T2-resistant but λ-sensitive bacteria, delaying the evolution of cross-resistance until after exposure to the second phage. Under this framework, the cocktail treatments—where both phages were present simultaneously—were expected to generate resistance dynamics intermediate between these extremes, as the selection for cross-resistance and single-phage resistance occurred concurrently. This hypothesis made explicit predictions about the timing and frequency of resistance and cross-resistance evolution, which we tested by directly sampling bacterial populations over time.

To track resistance evolution, we isolated bacterial clones from replicate populations subjected to the late dosing treatments (T2-first late, λ*trn*-first late, and cocktail late) in the initial experiment. From each replicate population, we attempted to isolate three independent colonies per sampling day from frozen community samples, allowing us to capture both within- and among-population variations in the resistance phenotypes. Because the assays used to quantify resistance and cross-resistance were labor-intensive, it was not feasible to sample all days of the experiment. Instead, sampling days were chosen based on observed population dynamics, focusing on periods when the bacterial populations transitioned from phage-mediated suppression to renewed growth—when resistance evolution was expected to be most informative.

Standard efficiency-of-plaquing (EOP) assays were not suitable for quantifying resistance in this system. Several evolved bacterial isolates displayed mucoidy or other cell-surface modifications that prevented reliable plating in soft agar overlays, while in other cases, phages produced zones of clearing without discrete plaques, precluding accurate plaque enumeration. Because EOP assays require countable plaques to quantify resistance, we instead assessed phage resistance using a liquid culture killing assay that measured short-term bacterial survival following phage exposure.

For each bacterial isolate, we quantified the bacterial density following a 30 min phage challenge in liquid culture—a time frame corresponding to a single round of infection with negligible phage population growth—and compared this to paired no-phage controls (see Section 2). The resistance was summarized using a resistance coefficient (RC), which compared the post-infection bacterial density of an evolved isolate to that of the ancestral *E. coli* strain under identical conditions. RC values of 0 indicated no change from ancestor, above 0 indicated an increased resistance, and below 0 was an increased sensitivity.

**Figure 4:**
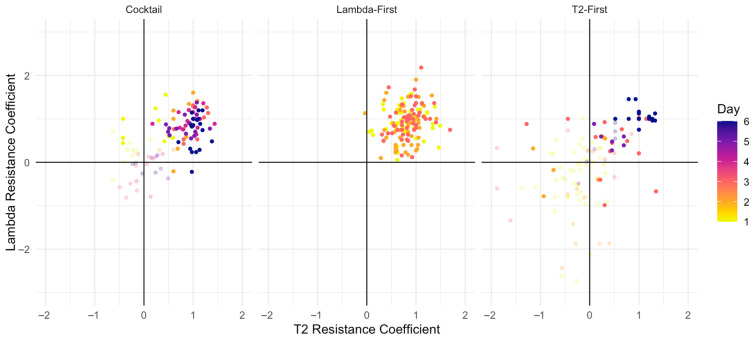
Cross-resistance profiles of bacterial isolates sampled across treatments and time points. Each point represents the resistance coefficients of a single isolate. Negative coefficients indicate increased sensitivity relative to the ancestral *E. coli* K-12 strain, whereas positive values indicate increased resistance. Transparent symbols denote isolates not significantly different from the ancestor, while opaque symbols indicate significance in at least one resistance dimension. Color gradient reflects the day on which the isolate was sampled, e.g., yellow indicates day 1, and blue/purple day 6. Cross-resistance evolved most rapidly in λ-first treatment, followed by cocktail, and then T2-first. See Supporting Information Figures S7–S12 for the dynamics of resistance broken down by treatment replicate and phage resistance.

In λ-first sequential treatments, the vast majority of isolates clustered in the cross-resistance quadrant, indicating increased resistance to both λ*trn* and T2 (Figure 4). Across all six replicate populations, bacteria evolved resistance to both phages within 24 h. Crucially, the resistance to T2 emerged prior to any exposure to T2, demonstrating that resistance evolved to λ*trn* conferred cross-resistance to T2. As a result, the second phage dose was ineffective, explaining the rapid loss of bacterial suppression and the elevated OD values observed in λ-first treatments.

Cross-resistance evolved most slowly and least frequently in the T2-first sequential treatments. No cross-resistant isolates were detected prior to exposure to λ*trn*, and cross-resistance first appeared on day 3, with the majority emerging on day 6. Moreover, cross-resistance arose in only three of six replicate populations, in contrast to the λ-first and cocktail treatments where it evolved in all populations. This delay and reduced frequency of cross-resistance coincided with sustained bacterial suppression.

T2-first treatments also produced several significant cases of collateral sensitivity, in which resistance to T2 was associated with increased susceptibility to λ*trn*, a few cases of collateral sensitivity in the reverse with λ*trn* resistance associated with increased T2 susceptibility, as well as numerous non-significant collateral sensitivity trends. Notably, we observed multiple significant cases of double sensitivity—increased susceptibility to both phages—early in the experiment. These instances corresponded to the four replicate populations that experienced early bacterial extinction, consistent with strong and persistent phage-mediated suppression.

In cocktail treatments, most isolates ultimately fell within the cross-resistance quadrant, indicating that simultaneous exposure to both phages can select for generalized resistance; however, both the magnitude and timing of this response were intermediately relative to the two sequential treatments. Cross-resistance emerged more slowly than in the λ-first treatments, appearing within ~48 h across replicate populations—approximately a 24 h delay—while remaining more pronounced than in the T2-first treatments, which showed the weakest tendency toward cross-resistance. Furthermore, some isolates from the T2-first treatment even exhibited the opposite response, collateral sensitivity, where increased resistance to T2 was coupled with increased sensitivity to λtrn. Taken together, these results indicated that the cocktail treatment did not generate a distinct resistance regimen but instead produced outcomes that fell between the two sequential extremes, consistent with the cocktail effectively behaving as an average of λ-first and T2-first selections rather than eliciting qualitatively novel evolutionary responses.

To evaluate the isolate-level resistance profiles and resistance evolution across treatments holistically, we analyzed all resistance coefficient (RC) data using a multilinear regression model. The model assessed the effects of the starting phage (T2, λ*trn*, or both), treatment type (sequential or cocktail), day of isolation, replicate population (tube), and test phage (T2 or λ*trn*) on RC values (Table S11). The analysis identified strong, significant direct effects of the starting phage (*p* = 2 × 10^−16^), day of isolation (*p* = 2 × 10^−16^), and replicate population (tube; *p* = 4.12 × 10^−10^) on resistance coefficients. These effects aligned with the treatment-specific resistance dynamics observed in the phenotyping assays. In particular, the λ-first sequential treatments consistently evolved cross-resistance within 24 h across all replicate populations, whereas the T2-first sequential treatments showed delayed and incomplete cross-resistance, first appearing on day 3 and arising in only half of the replicate populations. The significant tube effect reflected this within-treatment variability, which was most pronounced in the T2-first sequential treatments and included instances of collateral sensitivity.

Temporal effects were also evident. The significant effect of the day of isolation indicated that resistance phenotypes varied strongly over time, particularly in the cocktail and T2-first sequential treatments, where populations transitioned between susceptibility, single-phage resistance, and cross-resistance. Consistent with this, the model detected a significant phage-by-day interaction (*p* = 0.031), indicating that the resistance to T2 versus λtrn depended on when the isolates were sampled. Notably, the absence of a significant main effect of the test phage suggested that resistance frequently involved cross-resistance rather than phage-specific resistance.

Finally, a significant three-way interaction between the phage, day, and treatment type (*p* = 0.032) indicated that the evolution of cross-resistance depended jointly on delivery strategy and time. This interaction captured the distinct resistance trajectories observed across treatments, with the λ-first sequential treatments evolving cross-resistance earliest, the cocktail treatments evolving cross-resistance later, and the T2-first sequential treatments showing the greatest delay and variability.

### 3.4. Molecular Mechanisms Underlying Resistance and Cross-Resistance

To identify the genetic basis of resistance and cross-resistance across the treatments, we performed whole genome sequencing on three bacterial isolates from each treatment (Table S12). The isolates were selected to represent (i) the strongest cross-resistance phenotype, (ii) the earliest emergence of cross-resistance within each treatment, and (iii) distinctive resistance-associated phenotypes, such as mucoid colony formers observed in cocktails or collateral sensitivity observed in the T2-first sequential treatment.

All sequenced λ-first sequential isolates carried mutations in *envZ* (Table S13), a component of the EnvZ/OmpR two-component regulatory system [39] which can control expression of OmpF, a shared receptor used by both T2 and λ*trn*. Previous work has shown that wild-type *E. coli* K-12 grown in the experimental medium (‘Tris-LB’) expressed OmpF at substantially higher levels than LamB, the alternative λ*trn* receptor [40]. This expression pattern likely explained why resistance to λ*trn* evolved via modifications to OmpF rather than LamB. Because both phages used OmpF as a receptor, the mutations affecting OmpF regulation were expected to confer cross-resistance. Consistent with this expectation, the T2-first isolate exhibiting the strongest cross-resistance phenotype also harbored an *envZ* mutation, suggesting a shared genetic route to cross-resistance across treatments.

**Figure 5:**
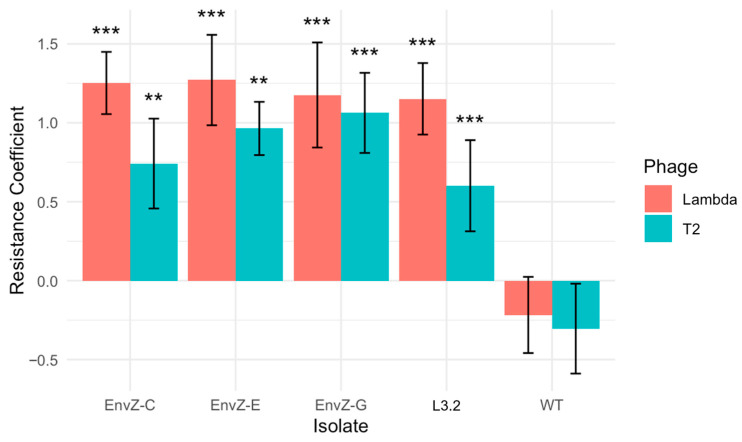
Resistance profiles for three strains independently edited with an EnvZ mutation (Q45P), the strain where the mutation was discovered in ‘L3.2’ and wild-type ‘WT’. Resistance was quantified using the resistance coefficient (RC) protocol. Error bars indicate 95% confidence intervals. Asterisks indicate estimates significantly different than the sensitive ancestor with *p* < 0.001 **, & *p* < 0.0001 ***.

Although *envZ* mutations evolved in all λ-first isolates and were associated with strong cross-resistance, such mutations have only rarely been observed in our laboratory [28], and their role in phage resistance has not been well characterized. To directly test whether *envZ* mutations conferred cross-resistance, we engineered the *envZ* mutation identified in the isolate λ-first day 1 ‘L3.2’ into an otherwise isogenic *E. coli* K-12 background using multiplex automated genome engineering (MAGE) [29]. Three independently engineered isolates were generated and assayed for resistance to both phages using the liquid culture survival assay. All engineered *envZ* mutants exhibited significantly increased resistance to both phages (Figure 5; T2 RC: ANOVA *p* = 3.14 × 10^−4^; λ*trn* RC: *p* = 5.23 × 10^−5^; Table S14), confirming that *envZ* mutations alone were sufficient to generate cross-resistance. The magnitude of resistance did not differ significantly from that of the original λ-first isolate in which the mutation arose (Table S15).

### 3.5. Mucoidy-Associated Cross-Resistance in Cocktail Treatments

The cocktail isolate with the strongest cross-resistance phenotype and exhibiting a mucoid colony phenotype (PCL R2.1; T2 RC = 1.0; λ*trn* RC = 1.4) carried two mutations: a premature stop codon in *igaA* and a mutation in *rcsD* (Table S13). Downregulation of *igaA* has been shown to increase bacterial fitness in the presence of both T2 and λ phages [41] implicating it in phage resistance. Although *igaA* is essential in *E. coli* [42], compensatory mutations in *rcsD* have been known to permit survival of otherwise lethal *igaA* disruptions [40,41]. We therefore inferred that the *igaA* mutation conferred cross-resistance, while the accompanying *rcsD* mutation acted as a compensatory mutation enabling viability. This mutational combination was previously shown to produce a mucoid phenotype [43,44]. Mucoidy can confer cross-resistance by increasing exopolysaccharide production, thereby physically obstructing phage access to surface receptors.

A second mucoid cocktail isolate carried a distinct mutation introducing a premature stop codon in *csrA*, resulting in a truncated protein. Previous studies in *E. coli* have shown that *csrA* mutants exhibited accelerated and enhanced biofilm formation across growth conditions [45], consistent with the observed mucoid phenotype and increased phage resistance.

### 3.6. Growth Costs Associated with Cross-Resistance Differ Among Treatments

Cross-resistance evolved more frequently in the cocktail and λ-first sequential treatments than in the T2-first sequential treatment. Cross-resistance arose in all replicate populations of both the cocktail (3/3) and λ-first (6/6) treatments, but in only half of the T2-first replicates (3/6). This pattern led us to hypothesize that cross-resistance in the T2-first treatment was associated with growth costs, making it less likely to emerge and persist, whereas cross-resistance in the cocktail and λ-first treatments was largely free of growth cost.

To test this hypothesis, we measured growth rates of representative bacterial isolates, alongside the ancestral wild-type strain, under conditions matching the original experiments. The growth trajectories were monitored over 24 h, and the maximum growth rates were compared among isolates.

**Figure 6:**
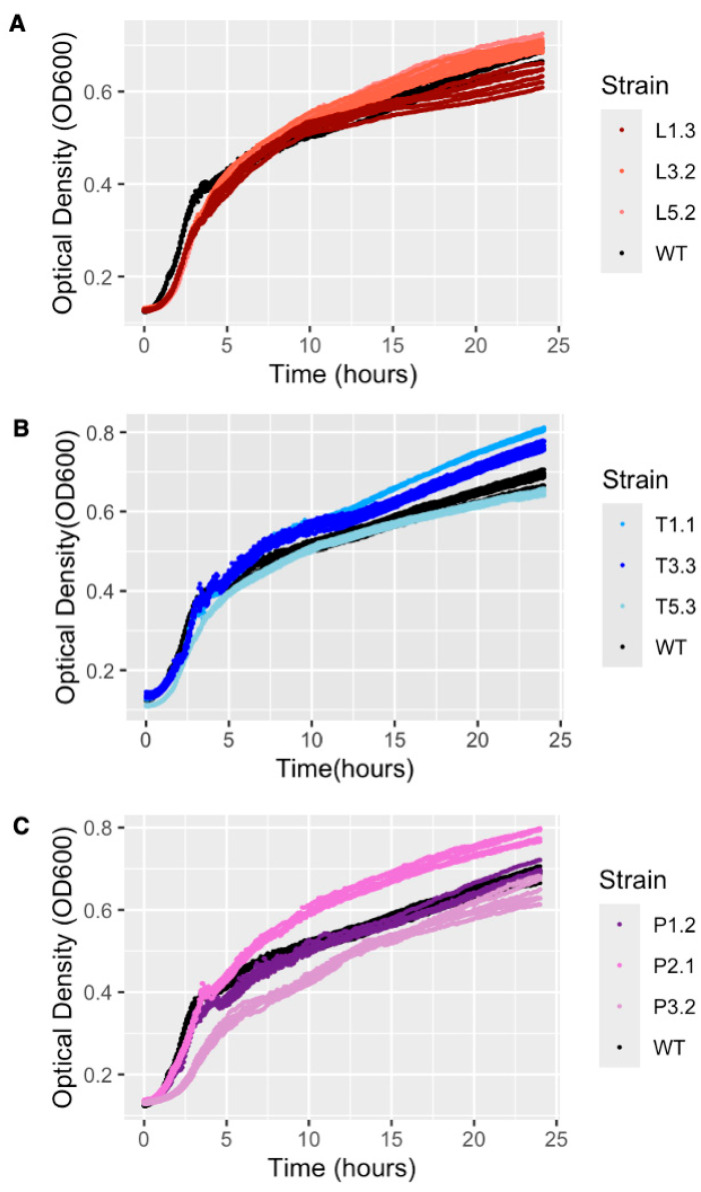
Growth curves showing bacterial density (OD_600_) measured over 24 h for individual isolates. (**A**) λ-first isolates plotted alongside wild-type (WT; black). (**B**) T2-first isolates plotted alongside WT (black). (**C**) Cocktail isolates plotted alongside WT (black). For some of the isolates we uncovered evolved mutations; L1.3: *envZ* T990G *acnB* T2045, L3.2: *envZ* T1423G, L5.2: *envZ* A1408G arc922_923 deletion, T1.1: *envZ* G368A, P2.1: *rcsD* C2165T *igaA* G1778, and P3.2 *csrA* C51A.

The growth rates differed significantly among isolates (Figure 6), (ANOVA; block 1: *p* = 4.28 × 10^−17^; block 2: *p* = 9.85 × 10^−4^) (Table S16). Cross-resistant isolates from the λ-first sequential treatments did not differ significantly in growth rate from the ancestral wild-type, indicating that cross-resistance in this treatment carried little or no growth cost. Similarly, the T2-first isolate carrying an *envZ* mutation—genetically similar to those observed in the λ-first isolates—also showed no detectable growth cost. This isolate was recovered from one of the few T2-first replicate populations that persisted throughout the experiment.

In contrast, growth costs were more variable among the isolates from the cocktail and T2-first treatments. The earliest collateral sensitivity isolate, T1.1 did not differ significantly from WT, while the strongest collateral sensitivity isolate showed a significantly higher growth rate than the ancestral strain. These results were consistent with a lack of growth costs, and a fitness advantage that may have contributed to its dominance prior to population extinction. For the T2-first and cocktail isolates, cross-resistance was primarily associated with mucoidy rather than *envZ* mutations. One mucoid isolate exhibited a significant reduction in growth rate (*p* = 3.6 × 10^−4^), whereas another showed no detectable growth cost relative to the ancestor.

While we did not directly study the growth of the engineered *envZ* mutant strains, we knew from the λ-first isolate L3.2, where the mutation was originally observed in isolation, that there were no detectable growth costs under conditions matching the original experiment. These results identified *envZ* mutations as no-growth-cost cross-resistance mutations. Together, these results indicated that cross-resistance can arise via multiple genetic and phenotypic routes that differ in their associated growth costs. Treatments that favored no-growth-cost cross-resistance (λ-first) promoted rapid and consistent resistance evolution, whereas treatments in which cross-resistance was costly or genetically constrained (T2-first and, variably, cocktails) delayed or limited its emergence.

## 4. Discussion

This study shows that the effectiveness of multi-phage therapy is determined less by the number of phages applied than by how treatment design structures the sequence and timing of selection experienced by bacterial populations. By directly comparing sequential and simultaneous phage exposure, we demonstrate that evolutionary outcomes are strongly path-dependent: the identity of the initial phage biases which resistance pathways are accessed and, in turn, whether bacteria rapidly evolve generalized cross-resistance or remain susceptible to subsequent phage challenge. These results provide a concrete experimental example of *evolutionary steering*, in which treatment order constrains future evolutionary options rather than simply summing selective pressures [46]. At the same time, our findings raise important questions about the mechanisms that generate this path dependence, the extent to which it can be predicted a priori, and how broadly these steering effects generalize across phages, hosts, and therapeutic contexts.

### 4.1. Cross-Resistance as a Key, but Not Exclusive, Constraint

Across treatments, the timing and frequency of cross-resistance emergence closely tracked treatment efficacy, reinforcing cross-resistance as a primary constraint on combination therapy. However, while we document clear differences in when and how often cross-resistance arises, we do not yet fully resolve *why* particular resistance routes are more accessible in some treatments than others. For example, the λ-first sequential treatments rapidly select for cross-resistance mediated by regulatory changes affecting shared phage receptors, whereas T2-first treatments delay or limit these outcomes. Whether these differences reflect intrinsic variation in treatment-specific population bottlenecks caused by differences in phage predation dynamics, or more subtle ecological feedback that shape which resistance mutations are favored remains an open question.

Cocktail treatments further highlight this complexity. When λtrn and T2 are applied simultaneously, outcomes are consistently intermediate between the λ-first and T2-first sequential treatments: cross-resistance evolves reliably, but neither as rapidly nor as uniformly as in the λ-first regime. At face value, this intermediate behavior is intuitive, reflecting concurrent selection by both phages; however, it is not a trivial expectation. If λ-driven selection alone is sufficient to rapidly generate high levels of generalized cross-resistance, then the cocktails should closely resemble the λ-first treatment, which they do not.

Instead, the cocktail treatments produce more heterogeneous resistance mechanisms, including the emergence of mucoidy with variable timing and fitness costs. These mucoid isolates suggest that simultaneous exposure broadens the set of accessible evolutionary responses, potentially by altering the relative strength, order, or ecological consequences of selection imposed by each phage. This pattern points to a role for T2-specific selection and eco-evolutionary feedback—such as density-dependent suppression, altered population structure, or shifts in the costs and benefits of envelope modification—that constrain or redirect the rapid evolution of λ-associated cross-resistance. How selection strength, standing genetic variation, and ecological context interact under simultaneous versus sequential exposure to favor regulatory versus structural resistance mechanisms remains unresolved, underscoring that cross-resistance is a dominant—but not exclusive—determinant of treatment outcome.

### 4.2. Rates, Costs, and Contingency in Resistance Evolution

Our results support the view that the evolutionary impact of resistance depends jointly on its accessibility and fitness consequences. Low-growth costs and repeatable resistance mechanisms promote rapid treatment failure, whereas costly routes can delay and lead to more heterogeneous outcomes. However, we only partially resolve how these factors interact; for example, cross-resistance arises through distinct genetic pathways—regulatory versus structural—but the conditions that favor one route over another remain unclear. Likewise, within-treatment variability, particularly in T2-first sequential populations, highlights the role of evolutionary contingency. Understanding when resistance outcomes are predictable versus historically contingent is an important open challenge for applying evolutionary steering in practice.

### 4.3. Uneven Phage Performance Influences Optimal Phage Delivery Strategy

While this has not yet been tested in other phage pairs, our results suggest a potentially generalizable principle for phage therapy design. When phages are unevenly matched in their ability to suppress bacterial populations, sequential administration beginning with the more suppressive phage may outperform simultaneous cocktail delivery. In such cases, adding a weaker phage concurrently may dilute the impact of the stronger phage, or as in this case, risk selecting for cross-resistance that compromises the more effective phage. We emphasize that this does not imply that phage cocktails are broadly inferior. Rather, cocktails may be most effective when constituent phages are more evenly matched in suppressive capacity. Our findings instead suggest a conditional rule: when one phage clearly outperforms another, deploying the stronger phage first and reserving the weaker as a secondary agent may maximize suppression while minimizing unintended resistance evolution. These results highlight the importance of assessing the individual phages prior to implementing phage therapy, either through cocktail or sequential administration.

### 4.4. Implications and Unanswered Questions for Phage Therapy Design

From a translational perspective, our findings caution against assuming that phage cocktails are universally optimal and highlight the potential of sequential strategies to exploit evolutionary steering. Yet important questions remain before such approaches can be broadly implemented: (1) How general are these dynamics across different phage–host systems, especially when phages do not share receptors or resistance pathways? (2) How sensitive are steering effects to dosing intervals, phage densities, or initial bacterial diversity? (3) And how do host immunity, spatial structure, and polymicrobial communities modify these evolutionary outcomes in vivo?

In addition, most clinical phage therapy is administered alongside antibiotics. Whether evolutionary steering can be amplified or disrupted by phage–antibiotic interactions, and whether sequential phage strategies can be integrated with standard antibiotic regimens, remain largely unexplored. Addressing these questions will be essential for translating laboratory insights into clinically actionable protocols.

## 5. Conclusions

Taken together, our results establish that treatment design can steer evolutionary outcomes in multi-phage therapy, with profound consequences for resistance evolution and treatment success. We show that sequential delivery can outperform cocktails by delaying or limiting cross-resistance, and we identify mechanistic differences that help explain these outcomes. At the same time, many questions remain about the generality, predictability, and mechanistic underpinnings of evolutionary steering in therapeutic contexts. By framing phage therapy as an evolutionary process rather than a static intervention, this study provides a foundation for future work aimed at understanding not only *whether* therapies fail, but *why*—and how those failures might be delayed or avoided.

## Data Availability

All experimental data is stored at Dryad doi:10.5061/dryad.stqjq2chf all Illumina sequencing reads are available on National Center for Biotechnology Information (NCBI) SRA: PRJNA1437269.

## Supplementary Materials

The following supporting information can be downloaded at: https://www.mdpi.com/article/10.3390/v18040404/s1, Figure S1: Phage titer in cocktail treatment by host, Figure S2: Phage titer in T2-first by host, Figure S3: Phage titer in *λ*-first by host, Figure S4: Phage titers in the *λ*-first late treatment on day 14 the experiment, measured on different host genotypes. Figure S5: Phage titers in the cocktail treatment on day 14 the experiment, measured on different host genotypes. Figure S6: Phage titers in the T2-first late treatment on day 14 the experiment, measured on different host genotypes. Figure S7: Time series plot showing the evolution of resistance to T2 in the T2-first bacterial populations in the first 6 days of the experiment. Figure S8: Time series plot showing the evolution of resistance to λtrn in the T2-first bacterial populations in the first 6 days of the experiment. Figure S9: Time series plot showing the evolution of resistance to T2 in the cocktail treatment bacterial populations in the first 6 days of the experiment. Figure S10: Time series plot showing the evolution of resistance to λtrn in the cocktail treatment bacterial populations in the first 6 days of the experiment. Figure S11: Time series plot showing the evolution of resistance to T2 in the λ-first treatment bacterial populations in the first 3 days of the experiment. Figure S12: Time series plot showing the evolution of resistance to λtrn in the λ-first treatment bacterial populations in the first 3 days of the experiment. Table S1: Experimental design of initial experiment. Table S2: Multiple linear regression modeling shows phage delivery strategy, dose timing, and starting phage shape treatment efficacy. Table S3: Multiple linear regression modeling focused on multi-phage treatment shows phage delivery strategy, dose timing, and starting phage shape treatment efficacy. Table S4: Pairwise comparison between WT-killing standard values from resistance assays shows T2 as a more suppressive phage than *λ*. Table S5: Pairwise comparisons show T2-first sequential treatments outperform cocktail treatments. Table S6: Pairwise comparisons show λ-first sequential treatments are outperformed by all other treatments. Table S7: Follow-up study design. Table S8: ANOVA comparisons among all three experimental runs shows no significant differences. Table S9: Pairwise comparisons show T2-first sequential treatments outperform cocktail treatments in follow-up studies. Table S10: Multiple linear regression modeling on shows phage delivery strategy and dose timing shape treatment efficacy in follow-up studies. Table S11: Multiple linear regression modeling shows day of isolation, starting phage, replicate population, phage administration method, and phage impact the evolution of resistance. Table S12: Bacterial isolates chosen for further analysis. Table S13: Sequencing results. Table S14: *EnvZ* mutants were significantly more resistant to both phages than WT. Table S15: Cross-resistance of *EnvZ* mutants was not significantly different from *λ*-first late isolate L3.2. Table S16: Growth rate analysis results. Table S17: AIC and R2 for the sigmoid relationship between the bacterial density by OD and CFU in the 14-day follow-up.

## Abbreviations

The following abbreviations are used in this manuscript:

RC	Resistance Coefficient
T2E	T2-First Early
T2M	T2-First Mid
T2L	T2-First Late
*λ*E	λ-First Early
*λ*M	λ-First Mid
*λ*L	λ-First Late
PCE	Phage Cocktail Early
PCM	Phage Cocktail Mid
PCL	Phage Cocktail Late
