# Supplementary material for: Sequential Phage Delivery Can Outperform Cocktails by Delaying Cross-Resistance Evolution
Source: Viruses. 2026 Mar 25;18(4):404. doi: 10.3390/v18040404 (PMC13119524; doi:10.3390/v18040404)
Supplement: Supplementary file 1 [file viruses-18-00404-s001.zip › viruses-4149377-supplementary.pdf]

Supplementary Figures

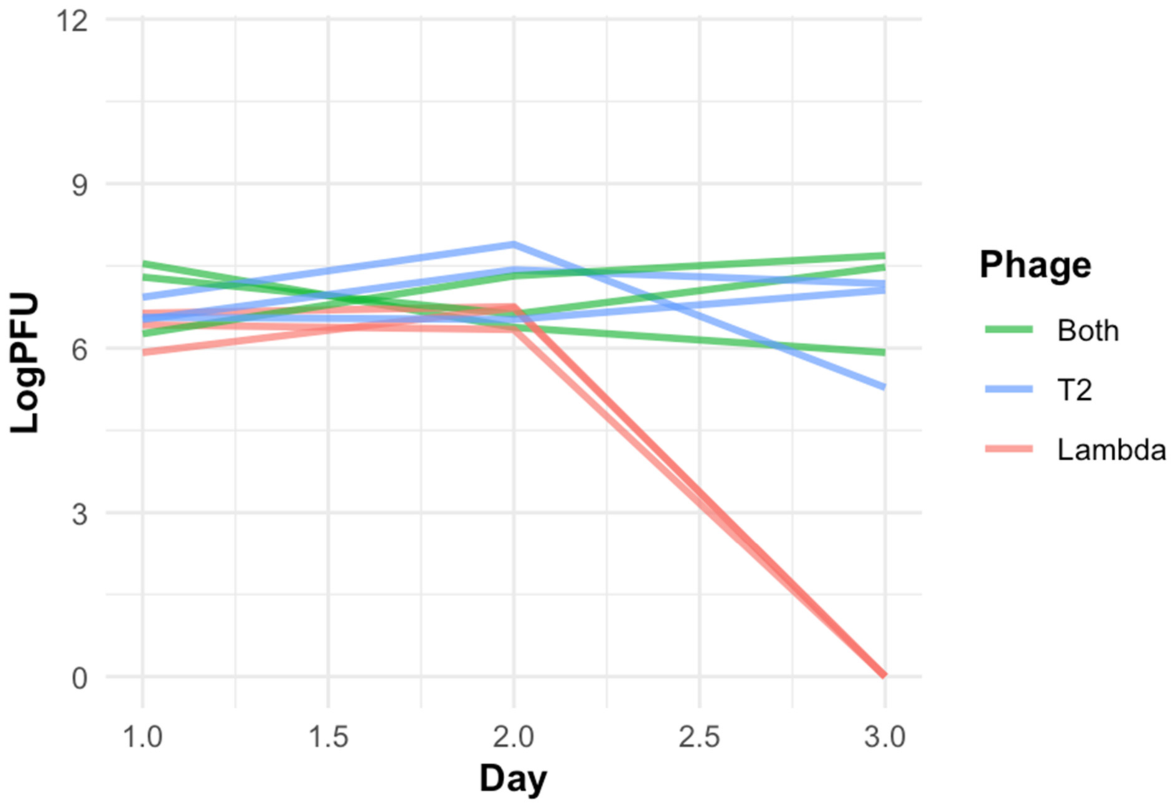

Figure S1. Log-transformed phage titers (LogPFU) in the Cocktail Late treatment during the first three days of the experiment, measured on different host genotypes. Frozen coculture samples from each replicate population were thawed and assayed by spot plating on three host strains: wild-type *E. coli* K-12, LamB⁻ OmpF⁻ *E. coli*, and FadL⁻ OmpF⁻ *E. coli*. T2 cannot infect FadL⁻ OmpF⁻ hosts, whereas *λ*trn cannot infect LamB⁻ OmpF⁻ hosts; both phages infect the wild-type strain. This differential plaquing allows enumeration of the presence and relative densities of each phage. These assays confirm that both phages were introduced at comparable densities and imposed selection for several days, during which the two phages initially coexisted. Notably, *λ*trn declined more rapidly than T2 as cross-resistance evolved (Figure 3).

Supplementary Figures

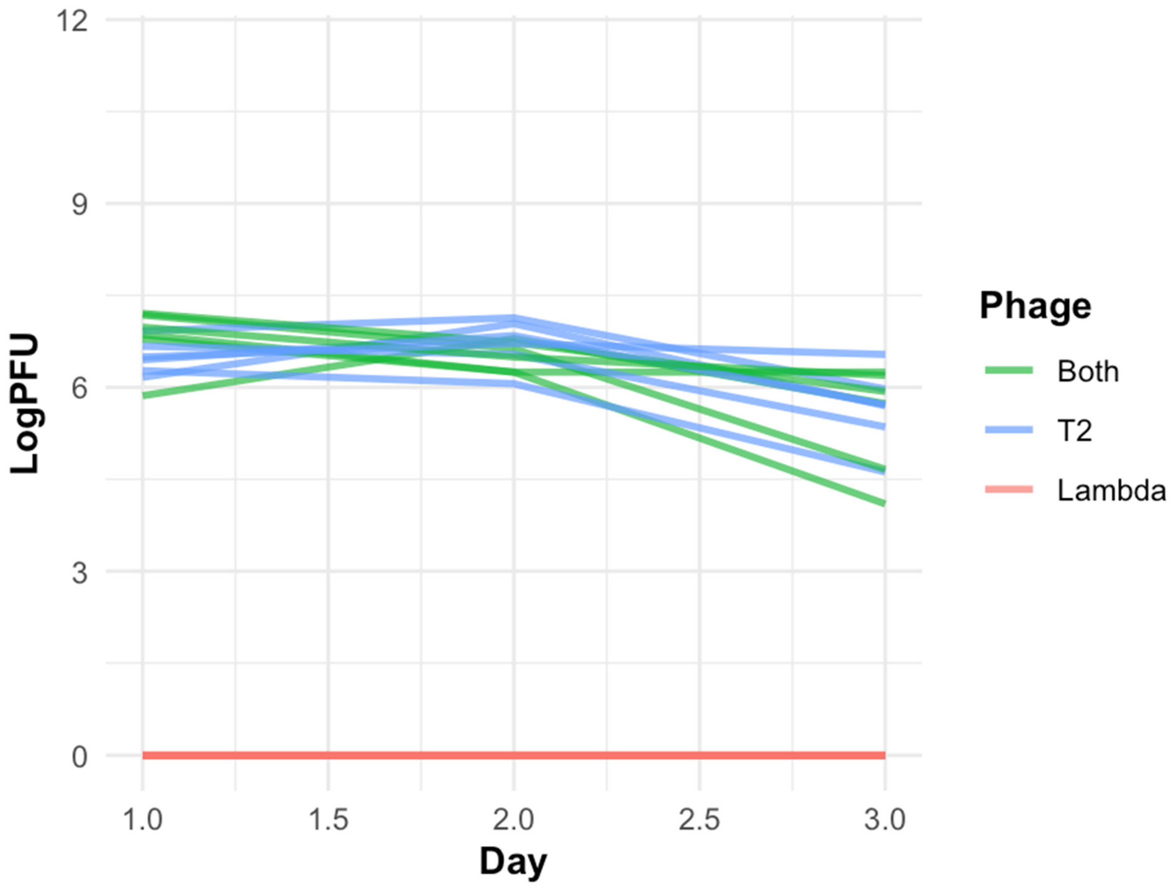

Figure S2. Log-transformed phage titers (LogPFU) in the T2-First Late treatment during the first three days of the experiment, measured on different host genotypes. Frozen coculture samples from each replicate population were thawed and assayed by spot plating on three host strains: wild-type *E. coli* K-12, LamB⁻ OmpF⁻ *E. coli*, and FadL⁻ OmpF⁻ *E. coli*. T2 cannot infect FadL⁻ OmpF⁻ hosts, whereas λtrn cannot infect LamB⁻ OmpF⁻ hosts; both phages infect the wild-type strain. These assays confirm that only T2 was present and imposing selection during the first three days of the experiment, prior to administration of the second phage dose.

Supplementary Figures

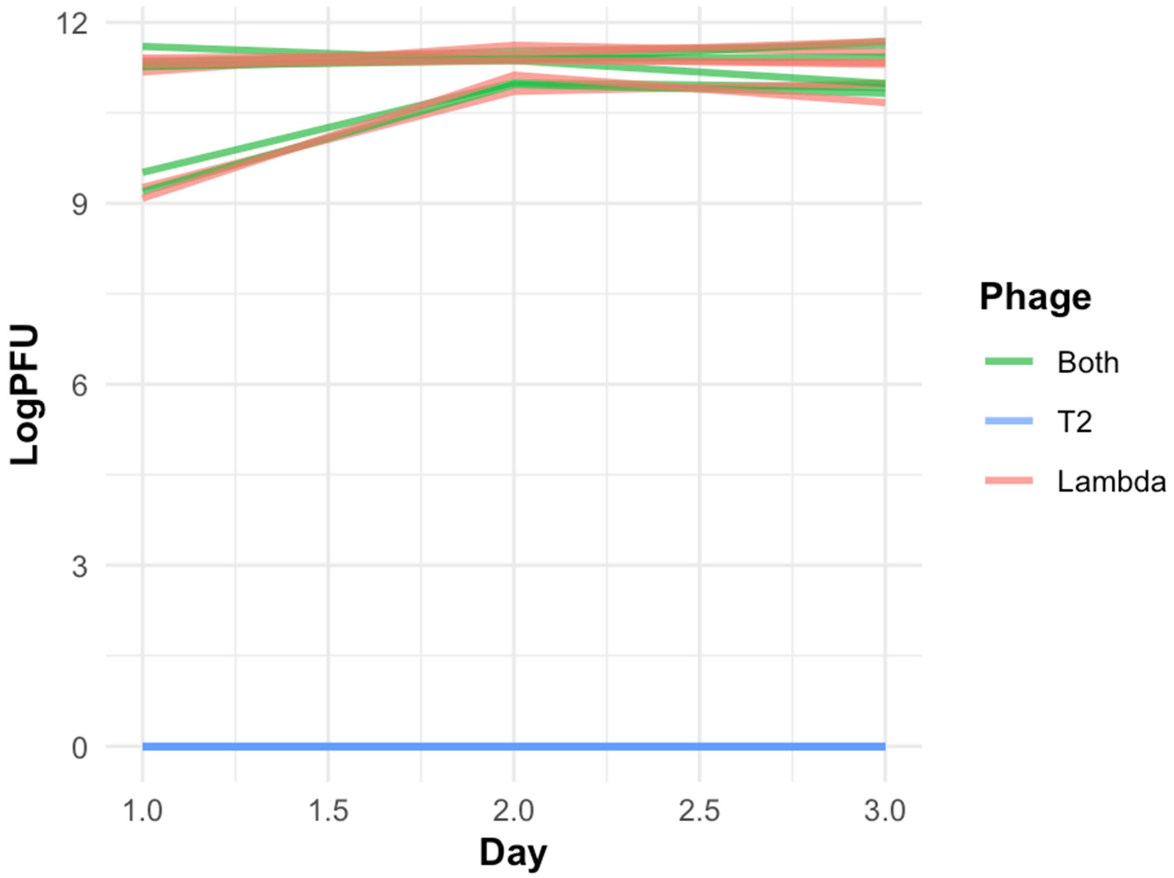

Figure S3. Log-transformed phage titers (LogPFU) in the λ-First Late treatment during the first three days of the experiment, measured on different host genotypes. Frozen coculture samples from each replicate population were thawed and assayed by spot plating on three host strains: wild-type *E. coli* K-12, LamB$^-$ OmpF$^-$ *E. coli*, and FadL$^-$ OmpF$^-$ *E. coli*. T2 cannot infect FadL$^-$ OmpF$^-$ hosts, whereas λtrn cannot infect LamB$^-$ OmpF$^-$ hosts; both phages infect the wild-type strain. These assays confirm that only λtrn was present and imposing selection during the first three days of the experiment, prior to administration of the second phage dose.

Supplementary Figures

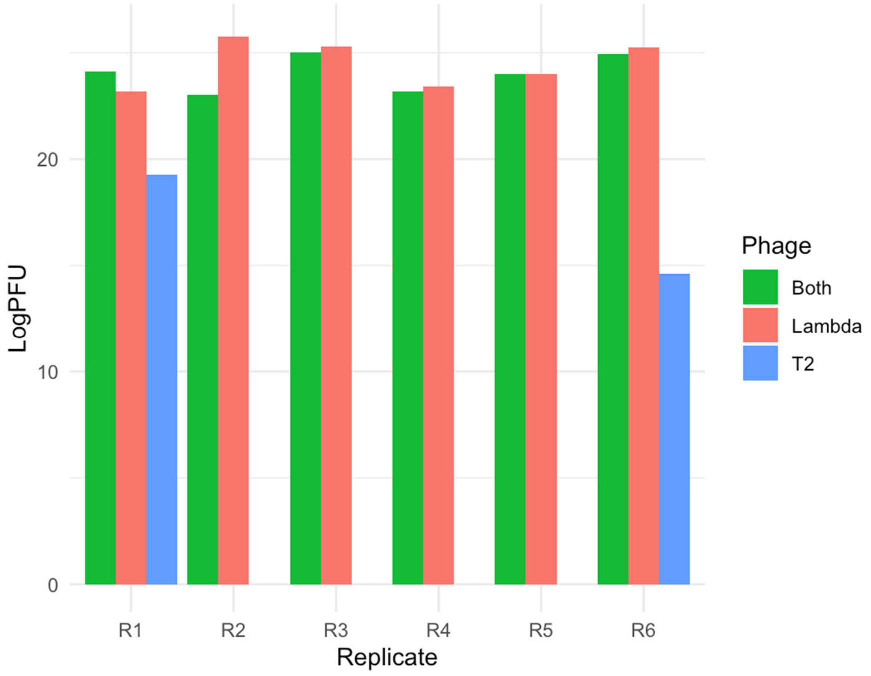

Figure S4. Phage titers in the λ-First Late treatment on day 14 the experiment, measured on different host genotypes. T2 titer shown in blue, λtrn in red and both phages in green. Frozen coculture samples from each replicate population were thawed and assayed by spot plating on three host strains: wild-type *E. coli* K-12, $LamB^{-}$ $OmpF^{-}$ *E. coli*, and $FadL^{-}$ $OmpF^{-}$ *E. coli*. T2 cannot infect $FadL^{-}$ $OmpF^{-}$ hosts, whereas λtrn cannot infect $LamB^{-}$ $OmpF^{-}$ hosts; both phages infect the wild-type strain. This shows that λtrn remained present throughout the experiment, while T2 persisted in only 2 of 6 replicates. This suggests that T2's reduced impact in this treatment, compared to when it is added initially, may be partly due to its loss—most likely caused by cross-resistance that evolved in this treatment.

Supplementary Figures

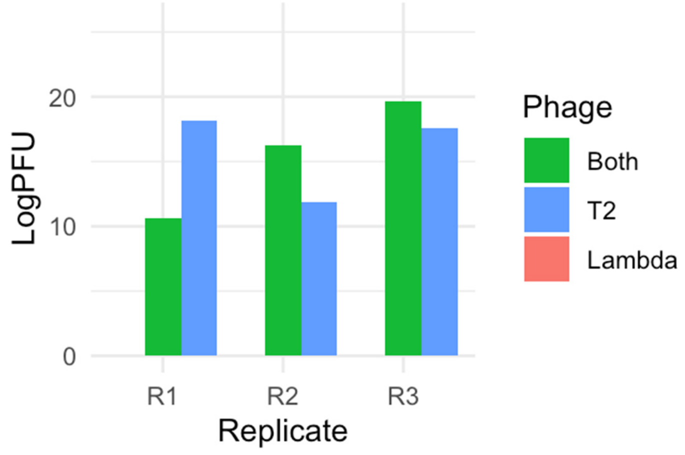

Figure S5. Phage titers in the Cocktail treatment on day 14 the experiment, measured on different host genotypes. T2 titer shown in blue, λtrn in red and both phages in green. Frozen coculture samples from each replicate population were thawed and assayed by spot plating on three host strains: wild-type *E. coli* K-12, LamB⁻ OmpF⁻ *E. coli*, and FadL⁻ OmpF⁻ *E. coli*. T2 cannot infect FadL⁻ OmpF⁻ hosts, whereas λtrn cannot infect LamB⁻ OmpF⁻ hosts; both phages infect the wild-type strain. These assays confirm that only T2 was present in the cocktail at day 14, consistent with figure S1 where λtrn went extinct between days two and three.T2 was maintained for the course of the experiment despite resistance eventually evolving and cell density rising.

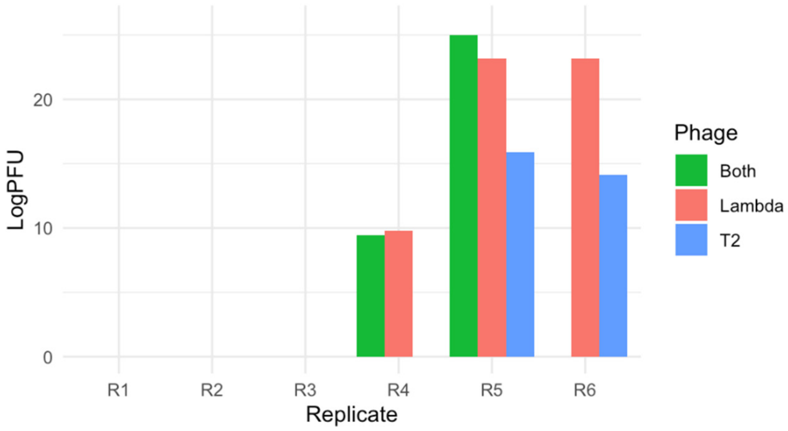

Figure S6. Phage titers in the T2-First Late treatment on day 14 the experiment, measured on different host genotypes. T2 titer shown in blue, λtrn in red and both phages in green. Frozen coculture samples from each replicate population were thawed and assayed by spot plating on three host strains: wild-type *E. coli* K-12, LamB⁻ OmpF⁻ *E. coli*, and FadL⁻ OmpF⁻ *E. coli*. T2 cannot infect FadL⁻ OmpF⁻ hosts, whereas λtrn cannot infect LamB⁻ OmpF⁻ hosts; both phages infect the wild-type strain. These results show there were phage extinction in some of the replicates, these occurred after the bacteria went extinct and are discussed in the main text. Additionally, in three replicates λtrn was maintained, and in two of those treatments T2 was also maintained.

Supplementary Figures

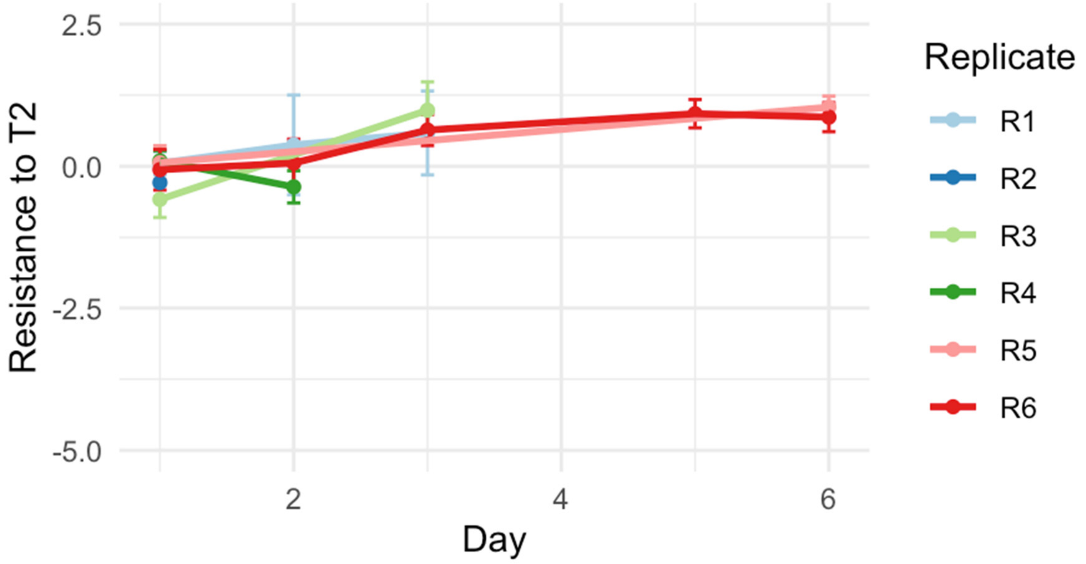

Figure S7. Timeseries plot showing the evolution of resistance to T2 in the T2-First bacterial populations in the first 6 days of the experiment. Each data point represents the average resistance coefficient (RC) of the three bacterial isolated from each replicate population. Error bars represent the 95% confidence interval. Missing data are from replicates where phage went extinct. Plot shows a gradual increase in resistance over six days with some within population heterogeneity observed on days two and three.

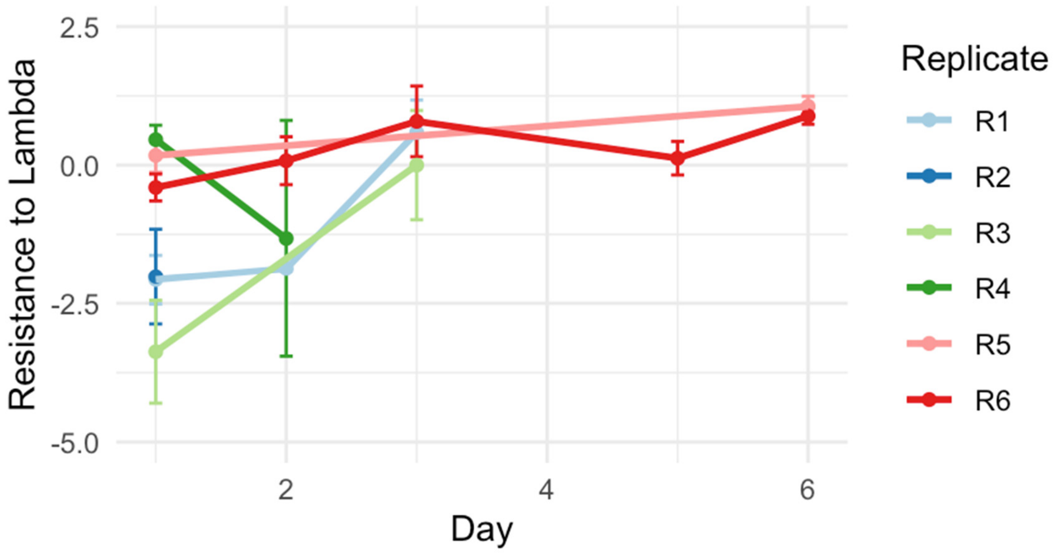

Figure S8. Timeseries plot showing the evolution of resistance to λtrn in the T2-First bacterial populations in the first six days of the experiment. Each data point represents the average resistance coefficient (RC) of the three bacterial isolated from each replicate population. Error bars represent the 95% confidence interval. Missing data are from populations where phage went extinct. Early time points show an increase in sensitivity to λtrn, then an eventual increase to resistance after λtrn was administered on day three. Within and between population genetic heterogeneity was observed in λtrn sensitivity at the early time points.

Supplementary Figures

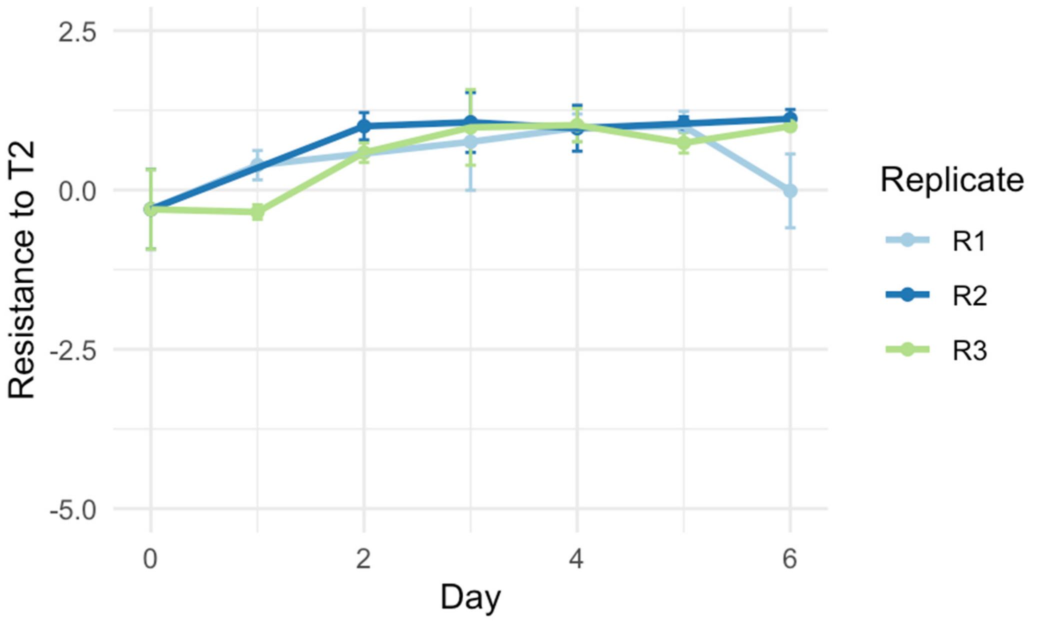

Figure S9. Timeseries plot showing the evolution of resistance to T2 in the Cocktail treatment in the bacterial populations in the first six days of the experiment. Each data point represents the average resistance coefficient (RC) of the three bacterial isolated from each replicate population. Error bars represent the 95% confidence interval.

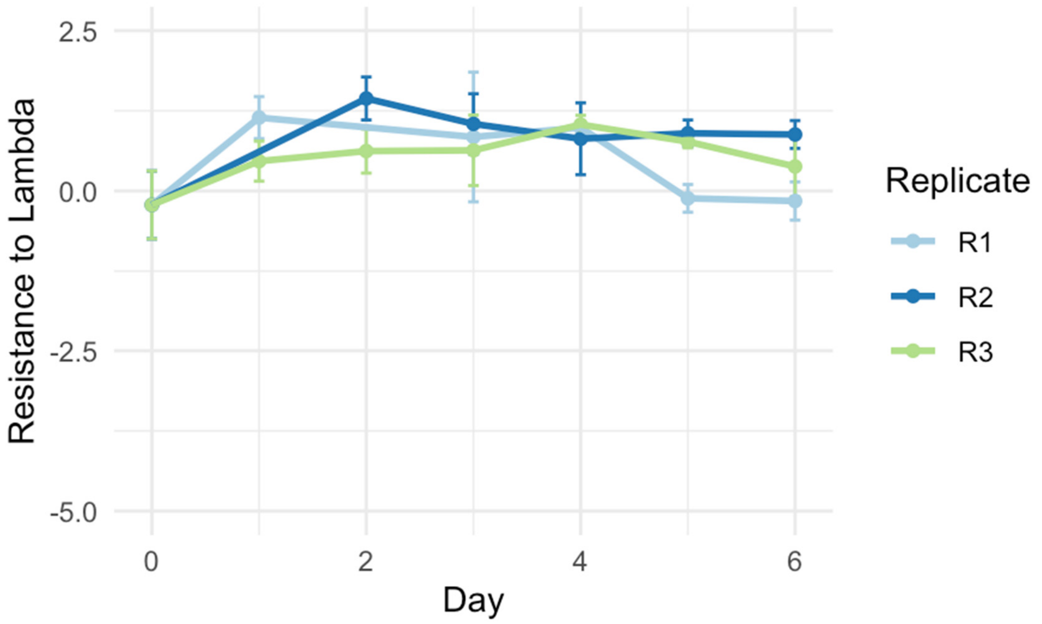

Figure S10. Timeseries plot showing the evolution of resistance to λtrn in the Cocktail treatment bacterial populations in the first 6 days of the experiment. Each data point represents the average resistance coefficient (RC) of the 3 bacterial isolated from each replicate population. Error bars represent the 95% confidence interval.

Supplementary Figures

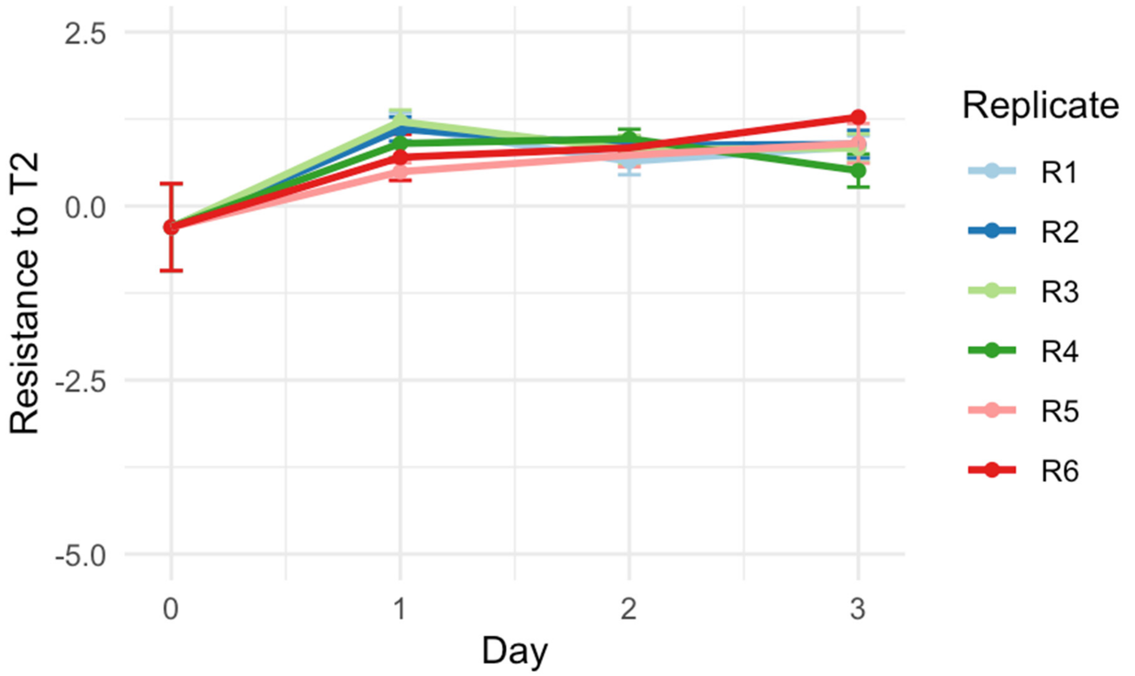

Figure S11. Timeseries plot showing the evolution of resistance to T2 in the λ-First treatment bacterial populations in the first 3 days of the experiment. Each data point represents the average resistance coefficient (RC) of the 3 bacterial isolated from each replicate population. Error bars represent the 95% confidence interval.

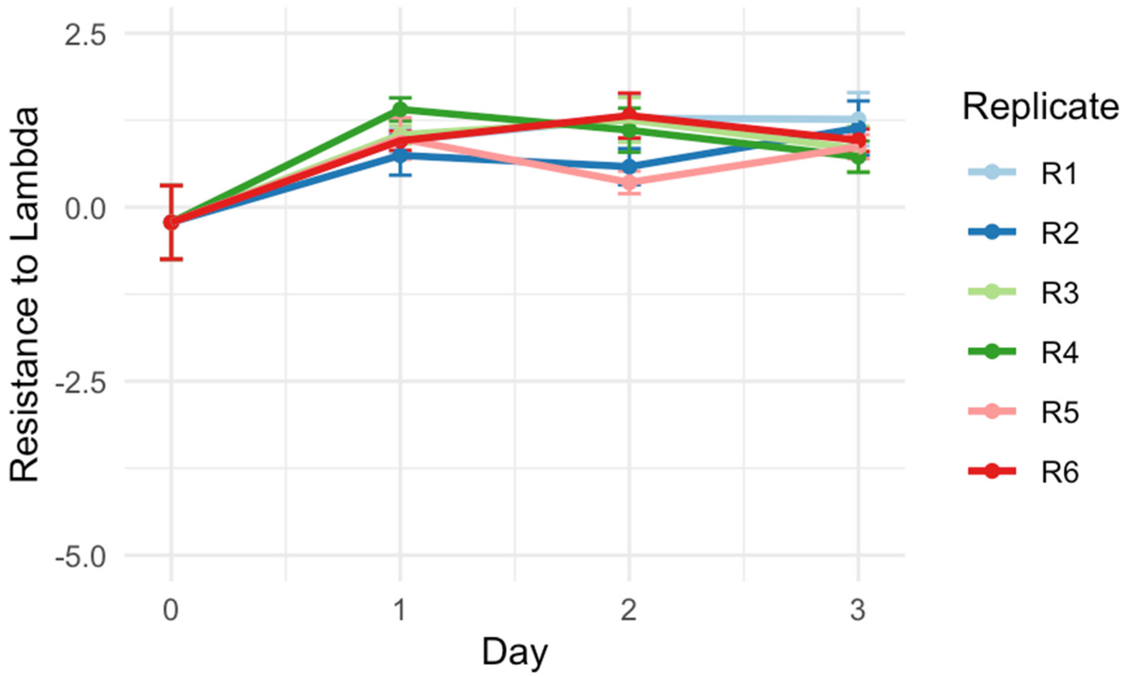

Figure S12. Timeseries plot showing the evolution of resistance to λtrn in the λ-First treatment bacterial populations in the first 3 days of the experiment. Each data point represents the average resistance coefficient (RC) of the 3 bacterial isolated from each replicate population. Error bars represent the 95% confidence interval.

Supplementary Tables

Table S1. Design of initial and most comprehensive suppression experiment.

Treatments	Abbreviation	2nd Dose?	2nd Dose Day	Starting Phage	Treatment Type	# Replicates
T2-First Early	T2E	yes	1	T2	Sequential	6
T2-First Mid	T2M	yes	2	T2	Sequential	6
T2-First Late	T2L	yes	3	T2	Sequential	6
λ-First Early	λE	yes	1	λtrn	Sequential	6
λ-First Mid	λM	yes	2	λtrn	Sequential	6
λ-First Late	λL	yes	3	λtrn	Sequential	6
Phage Cocktail Early	PCE	yes	1	Both	Cocktail	3
Phage Cocktail Mid	PCM	yes	2	Both	Cocktail	3
Phage Cocktail Late	PCL	yes	3	Both	Cocktail	3
T2 Single Phage Early	T2 SPE	yes	1	T2 only	Monophage	3
T2 Single Phage Mid	T2 SPM	yes	2	T2 only	Monophage	3
T2 Single Phage Late	T2 SPL	yes	3	T2 only	Monophage	3
λ Single Phage Early	λ SPE	yes	1	λtrn only	Monophage	3
λ Single Phage Mid	λ SPM	yes	2	λtrn only	Monophage	3
λ Single Phage Late	λ SPL	yes	3	λtrn only	Monophage	3
T2 Single	T2S	no	NA	T2 only	Monophage	3
λ Single	λS	no	NA	λtrn only	Monophage	3
No Phage	NP	no	NA	NA	Control	3

Details of the 18 treatments, including their names and abbreviations. The table indicates whether each treatment received a second phage dose and the timing of that second dose. Treatments are grouped into three broad categories: phage cocktails (abbreviated as "PC"), sequential treatments (named according to the starting phage and the timing of the second dose), and monophage treatments (single-phage treatments). Monophage treatments are further subdivided into two categories: two-dose monophage treatments and single-dose monophage treatments. The table also indicates the number of replicate populations for each treatment.

Supplementary Tables

Table S2. Multiple linear regression modeling shows phage delivery strategy, dose timing, and starting phage shape treatment efficacy

Model Terms	Options	Description
Type	Cocktail	Both phages given simultaneously
	Sequential	Phages given consecutively
	Monophage	Single phage given
Timing	Early	2nd dose of phage given 24h after 1st dose
	Mid	2nd dose of phage given 48h after 1st dose
	Late	2nd dose of phage given 72h after 1st dose
Starting Phage (SP)	T2	T2 phage given first
	λtrn	λtrn phage given first
	Both	T2 and λtrn given simultaneously
**RankOD~Type + Timing + SP + (Type * Timing * SP) + (Type * SP) + (Timing * SP)**		
**AIC**	**R2**	**$R2_{adj}$**
482.73	0.77	0.71
Type p = 0.006		
Starting Phage(SP) p = 3.29×10^{-15}		
Type*Starting Phage =0.014		
Type * Timing * Starting Phage p = 0.017		

Effects of phage therapy administration method, timing, and starting phage identity on bacterial density. Bacterial suppression varied significantly across treatment types (p = 0.006) and starting phage identity (p < 0.001). A significant interaction between treatment type and starting phage (p = 0.014), as well as a three-way interaction with timing (p = 0.017), indicates that treatment efficacy depended on both when therapy was administered and which phage was applied first.

Supplementary Tables

Table S3. Multiple linear regression modeling focused on multiphage treatment shows phage delivery strategy, dose timing, and starting phage shape treatment efficacy

Model Terms	Options	Description
Type	Cocktail	Both phages given simultaneously
	Sequential	Phages given consecutively
	Monophage	Single phage given
Timing	Early	2nd dose of phage given 24h after 1st dose
	Mid	2nd dose of phage given 48h after 1st dose
	Late	2nd dose of phage given 72h after 1st dose
Starting Phage (SP)	T2	T2 phage given first
	λtrn	λtrn phage given first
	Both	T2 and λtrn given simultaneously
**RankOD~Type + Timing + SP + (Type * Timing * SP) + (Type * SP) + (Timing * SP)**		
**AIC**	**R2**	**$R2_{adj}$**
308.52	0.79	0.74
Type p = 0.007		
Starting Phage(SP) $p = 1.091 \times 10^{-12}$		
Timing*Type p = 0.06		
Timing*Starting Phage (SP) p = 0.068		

Effects of multiphage therapy administration method, timing, and starting phage identity on bacterial density (RankOD). Bacterial suppression varied significantly across treatment types (p = 0.007) and starting phage identity (p < 0.001). A near-significant interaction between treatment timing and starting phage (p = 0.060, as well as an interaction with timing & type (p = 0.068), indicates possible trend where treatment efficacy is impacted by both when therapy was administered, by which method and which phage was applied first.

Supplementary Tables

Table S4. Pairwise comparison between WT-killing standard values from resistance assays shows T2 is a more suppressive phage than λ

Phage	Mean WT Benchmark	N
T2	-7.17	22
Lambda	-5.62	20
t test p value	0.022	

Pairwise comparison between the resistance coefficient WT benchmark , $ln(WT_P / WT_{NP})$, of each phage shows that T2 is a more suppressive phage under our experimental conditions.

Table S5. Pairwise comparisons show T2-First Sequential treatments outperform cocktail treatments

Comparison	Day After 2nd Dose	Test	p value	Lower OD
T2E & PCE	2	Mann-Whitney	0.428	NA
T2M & PCM	3	t test	0.004	T2M
T2L & PCL	4	t test	0.024	T2L

Pairwise comparisons between cocktail treatments; Phage Cocktail Early (PCE), Phage Cocktail Mid (PCM), & Phage Cocktail Late (PCL) and sequential treatments starting with T2; T2-First Early (T2E), T2-First Mid (T2M), & T2-First Late (T2L). Comparisons made between bacterial density (OD) 24 hours after the second dose was administered. Sequential treatments outperformed the cocktail treatments in all cases where significant differences were seen.

Table S6. Pairwise comparisons show λ-First Sequential treatments are outperformed by all other treatments

Comparison	Day	Test	p value	Lower OD
T2E & λE	2	Mann-Whitney	0.001	T2E
T2M& λM	3	Mann-Whitney	0.005	T2M
T2L& λL	4	Mann-Whitney	0.005	T2L
T2L & T2-Only	4	t test	0.0267	T2L
PCE & λE	2	Mann-Whitney	0.028	PCE
PCM & λM	3	Mann-Whitney	0.028	PCM
PCL & λL	4	Mann-Whitney	0.028	PCL
PCL & T2-Only	4	t test	0.789	NA
λL & λ-Only	4	Mann-Whitney	0.519	NA

Showing pairwise comparisons between the λtrn sequential treatments: λ-First Early (λE), λ-First Mid (λM), λ-First Late (λL) and the other treatments: T2-First Early (T2E), T2-First Mid (T2M), T2-First Late (T2L), & cocktail treatments: Phage Cocktail Early (PCE), Phage Cocktail Mid (PCM) and Phage Cocktail Late (PCL). Comparisons made between bacterial density (OD600) 24 hours after the 2nd dose of phage was given. All other treatments starting outperformed those starting with λ regardless of timing.

Supplementary Tables

Table S7. Follow-up study design

Treatment	Abbreviation	2nd Dose Day	Starting Phage	Treatment Type	#Replicates
T2-First Mid	T2M	2	T2	Sequential	12
T2-First Late	T2L	3	T2	Sequential	12
Phage Cocktail Mid	PCM	2	Both	Cocktail	12
Phage Cocktail Late	PCL	3	Both	Cocktail	12

Details of the follow up studies; the treatments, their names, abbreviations, dosing schedule, starting phage, treatment type and number of replicates.

Table S8. ANOVA comparisons among all three experimental runs shows no significant difference

Treatment	ANOVA p value
T2M	0.146
T2L	0.892
PCM	0.999
PCL	0.725
All	0.465

ANOVA comparisons among the initial experiment, the 5-day follow up, and the 14-day follow up show no significant difference in OD across experimental runs. The mean bacterial density for each replicate population averaged across the full 14-day experiment was used for this analysis

Table S9. Pairwise comparisons show T2-First Sequential treatments outperform cocktail treatments in follow up studies

Experiment	Duration	Comparison	Test	p value	Lower OD
5-Day Follow Up	1-5	T2M & PCM	Mann-Whitney	0.019	T2M
5-Day Follow Up	1-5	T2L & PCL	Mann-Whitney	0.006	T2L
14-Day Follow Up	1-14	T2M & PCM	Mann-Whitney	0.488	NA
14-Day Follow Up	1-14	T2L & PCL	Mann-Whitney	0.035	T2L

Pairwise comparisons between the sequential; T2-First Mid (T2M) & T2-First Late (T2L) and the cocktail treatments; Phage Cocktail Mid (PCM) & Phage Cocktail Late (PCL) in the 2 follow up studies. The 1st follow up was done for 5 days, and the 2nd was done for 14 days. The sequential treatments outperformed the cocktail treatments in all comparisons where there were significant differences (5-day T2M &PCM $p = 0.019$, T2L & PCL $p = 0.006$), (14- day T2L & PCL $p = 0.035$).

Supplementary Tables

Table S10. Multiple linear regression modeling shows phage delivery strategy, and dose timing shape treatment efficacy in follow up studies

**Model Terms**	**Options**	**Description**
Type	Cocktail	Both phages given simultaneously
	Sequential	Phages given consecutively
Timing	Mid	2nd dose given 48h after 1st
	Late	2nd dose given 72h after 1st
**14-Day: RankOD~Type + Timing + Type * Timing)**		
**AIC**	**R2**	**$R2_{adj}$**
479.01	0.53	0.50
Type p = 3.04×10^{-9}		
Type * Timing p = 0.031		
**5-Day: RankOD~ Timing**		
**AIC**	**R2**	**$R2_{adj}$**
384.13	0.26	0.21

Effects of phage therapy administration method (Cocktail or Sequential), and timing (Mid or Late) seen in the 14 day, and 5 day follow up studies. Bacterial suppression varied significantly between treatment types (14d: type p = <0.001, 5d type p < 0.001), and there was a significant interaction between treatment type and timing (14d: type * timing p = 0.03) indicating that suppression can be impacted by both the type of phage therapy as well as when phages are given.

Supplementary Tables

Table S11. Multiple linear regression modeling shows day of isolation, starting phage, replicate population, phage administration method, and phage impact the evolution of resistance.

**Model Terms**	**Options**	**Description**
Treatment	Cocktail<br>Sequential<br>Monophage	Both phages given simultaneously<br>Phages given consecutively<br>Single phage given
Day	Days 0-6	Day of experiment the bacteria was isolated from
Phage	T2 or λtrn	Phage the bacteria is being tested for resistance to
Tube	C1-3 (Cocktail population 1-3)<br>SL1-6 (λ-First population 1-6)<br>ST1-6 (T2-First population 1-6)	Replicate population from which the bacteria was isolated
Starting Phage (SP)	T2<br>λtrn<br>Both	T2 phage given first<br>λtrn phage given first<br>T2 λtrn given simultaneously
**RankRC~ Phage + SP + Day + Treatment + Phage * Day + Phage * Tube + SP * Day + SP * Treatment + SP * Tube + Day * Treatment + Day * Tube + Phage * Treatment * SP + Phage * Day * Treatment + Phage * Treatment * Tube + Phage * SP * Tube + Phage * Treatment * Tube**		
AIC	R2	$R2_{adj}$
11575	0.50	0.46
Starting Phage(SP) $p < 2.0 \times 10^{-16}$		
Day $p < 2.0 \times 10^{-16}$		
Tube $p = 4.747 \times 10^{-7}$		
Phage * Day p = 0.044		
Phage* Tube p = 0.027		
Starting Phage (SP) * Day p = 0.006		
Day * Tube $p = 6.0 \times 10^{-4}$		
Phage * Day * Treatment p = 0.028		
Phage*Day*Tube p = 0.029		

Effects of multiphage therapy administration method, time (day), starting phage, replicate population (tube), and phage being tested for resistance (phage) on the evolution of phage resistance seen in the initial 14-day experiment. The starting phage (p < 2e-16), time (day p < 2e-16) and tube (the replicate population from which the bacteria was isolated, p = 4.747e-07) have significant direct effects on the resistance coefficient. The interaction between phage and day (p = 0.044), phage and tube (p = 0.027), starting phage and day (p. = 0.006), day and tube (p < 0.001) have significant impact on resistance as quantified by the RC score. There is a significant triple interaction between phage, day and treatment type (p = 0.03), and between phage, day and tube (p = 0.028).

Supplementary Tables

Table S12. Bacterial isolates chosen for further analysis

Treatment	Isolation Day	Replicate	Colony	Isolate ID	Phenotype	T2 RC	λRC
λ-First Late	3	R1	3	L1.3	Strongest cross resistance	0.99	1.87
λ-First Late	1	R3	2	L3.2	Earliest cross resistance	1.27	1.15
λ-First Late	3	R5	2	L5.2	Equal cross resistance	0.92	0.95
Cocktail Late	1	R1	2	P1.2	Earliest cross resistance	0.51	0.54
Cocktail Late	2	R2	1	P2.1	Strongest cross resistance	1	1.44
Cocktail Late	3	R3	3	P3.2	Mucoidy	1.08	1.1
T2-First Late	2	R1	1	T1.1	Earliest collateral sensitivity	0.38	-1.87
T2-First Late	6	R5	3	T5.3	Strongest cross resistance	1.24	1.34
T2-First Late	3	R3	3	T3.3	Collateral sensitivity	0.67	-0.57
WT *K12 E. coli*	0	NA	NA	WT	Ancestral bacteria	-0.3	-0.22

Bacterial isolates chosen for further analysis, their phenotypes and resistance coefficient (RC) scores for both phages.

Supplementary Tables

Table S13. Bacterial mutations revealed through whole genome sequencing

Bacterial Isolate	Day of Isolation	Sequencing ID	Phenotype	Mutation position	Nucleotide Change	Annotation	Gene
λ-First Late	1	L1.3	Strongest cross resistance	3,528,380	**T→G**	**Q283P (CAG→CCG)**	*envZ* ←
λ-First Late	3	L3.2	Earliest cross resistance	3,529,094	**T→G**	**Q45P (CAG→CCG)**	*envZ* ←
λ-First Late	3	L5.2	Equal cross resistance	3,529,079	**A→G**	**L50P (CTC→CCC)**	*envZ* ←
λ-First Late	3	L5.2	Equal cross resistance	480,803	****(GA)$_{4\rightarrow3}$**	**coding (272-273/1194)nt**	*acrA* ←
Phage Cocktail Late	2	P2.1	Earliest cross resistance	2,309,131	**C→T**	**T722I (ACA→ATA)**	*rcsD* →
Phage Cocktail Late	2	P2.1	Strongest cross resistance	3,521,605	**G→A**	**W593* (TGG→TAG)**	*igaA* →
Phage Cocktail Late	3	P3.2	Mucoidy	2,812,370	**C→A**	**E46* (GAG→TAG)**	*csrA* ←
T2-First Late	6	T5.3	Strongest cross resistance	3,528,039	**G→A**	**R397C (CGC→TGC)**	*envZ* ←

Showing the bacterial isolates sequenced, why they were chosen for sequencing and the mutations found. **(GA) $_{4\rightarrow3}$ indicates that L5.2 has 3 (GA) repeats rather than the 4 (GA) repeats found in the WT. The * in W593* & E46* indicates that the mutation results in a truncated protein.

Supplementary Tables

Table S14. *EnvZ* mutants are significantly more resistant to both phages than WT is.

**ANOVA RC p =3.14 × 10^{-4}(T2), p= 5.23 × 10^{-5}(λtrn)**			
**Bacteria**	**Phage**	**Average RC**	**Tukey's HSD (Compared to WT)**
WT	T2	-0.30	NA
WT	λtrn	-0.22	NA
L3.2	T2	0.60	3.0 X 10^{-4}
L3.2	λtrn	1.15	2.0 X 10^{-4}
EnvZ-C	T2	0.74	0.003
EnvZ-C	λtrn	0.97	1.0 X 10^{-4}
EnvZ-E	T2	0.96	0.001
EnvZ-E	λtrn	1.80	1.0 X 10^{-4}
EnvZ-G	T2	1.10	8.0 x 10^{-4}
EnvZ-G	λtrn	0.90	2.0 x 10^{-4}

EnvZ mutants are significantly more resistant to both phages than WT is, as shown by the resistance coefficient (RC). The *envZ* mutation was first observed in the λ-First Late isolate L3.2

Supplementary Tables

Table S15. Cross resistance of *EnvZ* mutants is not significantly different from λ-First Late isolate L3.2.

ANOVA RC p =3.14 × 10^{-4}(T2), p= 5.23 × 10^{-5}(Lambda)			
**Bacteria**	**Phage**	**Average RC**	**Tukey's HSD (Compared to L3.2)**
L3.2	T2	0.60	NA
L3.2	λtrn	1.15	NA
EnvZ-C	T2	0.74	0.37
EnvZ-C	λtrn	0.97	0.96
EnvZ-E	T2	0.96	0.73
EnvZ-E	λtrn	1.80	0.93
EnvZ-G	T2	1.10	0.91
EnvZ-G	λtrn	0.90	0.99

Cross resistance of *envZ* mutants is not significantly different from λ-First Late isolate L3.2. Resistance coefficient (RC) was used to quantify the phage resistance

Supplementary Tables

Table S16. Growth rate analysis results

Treatment	Day	Replicate	Colony	Isolate ID	Block	**μMax**	Tukey's HSD p	Significance
T2-First Late	3	R3	3	T3.3	2	2.44 x 10^{-4}	7.5 x 10^{-3}	Higher
T2-First Late	6	R5	3	T5.3	2	1.63 x 10^{-4}	0.609	NA
T2-First Late	2	R1	1	T1.1	3	1.89 x 10^{-4}	0.129	NA
Phage Cocktail Late	3	R3	2	P3.2	1	1.04 x 10^{-4}	4.0 x 10^{-4}	Lower
Phage Cocktail Late	2	R2	1	P2.1	1	**1.35** x 10^{-4}	0.931	NA
Phage Cocktail Late	1	R1	2	P1.2	1	1.64 x 10^{-4}	0.236	NA
λ-First Late	3	R5	2	L5.2	1	1.47 x 10^{-4}	0.999	NA
λ-First Late	1	R3	2	L3.2	1	1.48 x 10^{-4}	1	NA
λ-First Late	3	R1	1	L1.3	1	1.39 x 10^{-4}	0.999	NA
WT *K12 E. coli*	0	NA	NA	WT	1-3			
ANOVA Block 1	1.97 x 10^{-4}			WT Block 1		1.44 x 10^{-4}		
ANOVA Block 2	9.85 x 10^{-4}			WT Block 2		1.80 x 10^{-4}		
ANOVA Block 3	0.110			WT Block 3		1.45 x 10^{-4}		

Shows average maximum growth rates (**μMax**) for each bacterial isolate, the results of ANOVA analysis comparing the maximum growth rates, and the Tukey's HSD post hoc analysis results. The collateral sensitivity isolate from T2-Sequential had a significantly higher max growth rate than WT ($p = 0.003$), while ½ of the cross resistant isolates from this treatment had significantly lower max growth rate (T5.2 $p = 3.95e-10$). All λ-Sequential isolates showed no significant differences in growth rate compared to WT. The mucoidy cocktail isolate (P3.2 $p < 0.001$) had a significantly lower max growth rate compared to WT. The cross resistant cocktail isolates (P1.2, P2.1) showed no significant differences in growth rate.

Supplementary Tables

Table S17. Model comparison for the relationship between bacterial density measured by optical density (OD) and colony-forming units (CFU) using samples spread throughput the 14-day follow-up experiment. Data were analyzed separately for each treatment to account for potential treatment-specific differences. 156 datapoints were used for each trial. Multiple model forms were evaluated, including linear, quadratic, power, exponential, von Bertalanffy, Michaelis–Menten, logistic (sigmoid), and Gompertz functions. The logistic (sigmoid) model provided the best fit across treatments based on AIC and yielded high R^2values. Given this strong correspondence between OD and CFU, and the practical efficiency of measuring OD, most bacterial density measurements in the study were quantified using optical density.

Treatment	AIC	R2
TM	15.78	0.75
PCM	11.37	0.84
T2L	8.92	0.83
PCL	11.89	0.89
